# A short-acting psychedelic intervention for major depressive disorder: a phase IIa randomized placebo-controlled trial

**DOI:** 10.1038/s41591-025-04154-z

**Published:** 2026-02-16

**Authors:** David Erritzoe, Tommaso Barba, Tiffanie Benway, Zelah Joel, Meghan Good, Marie Layzell, Michelle Baker Jones, Graham Campbell, Ashleigh Murphy-Beiner, Peter Rands, Malcolm Boyce, Helen Topping, Brandon Weiss, Christopher Timmermann, David Nutt, Robin Carhart-Harris, Carol Routledge, Ellen James

**Affiliations:** 1 Imperial College London, London, UK; 2 Formerly Cybin/Small Pharma, London, UK; 3 Cybin UK/Cybin IRL, London, UK; 4 MBJ Counselling, Kingston, UK; 5 HMR, London, UK; 6 Center for Psychedelic and Consciousness Research, Department of Psychiatry and Behavioral Sciences, Johns Hopkins School of Medicine, Baltimore, MD USA; 7 Department of Experimental Psychology, University College London, London, UK; 8 University of California, San Francisco, San Francisco, CA USA

**Keywords:** Depression, Trauma

## Abstract

Major depressive disorder (MDD) is a leading cause of disability worldwide, yet many patients have inadequate responses to current treatments. Dimethyltryptamine (DMT), a serotonergic psychedelic with rapid onset and short duration, shows promise as a potential antidepressant (AD), although clinical evidence in MDD remains limited. We conducted a phase IIa, double-blind, placebo-controlled, randomized clinical trial to evaluate the efficacy and safety of intravenous DMT (SPL026; DMT fumarate) in adults with moderate-to-severe MDD. Participants received a single 21.5-mg dose of DMT or placebo infused over 10 min, along with supportive psychotherapeutic support, followed by a 2-week assessment. A subsequent open-label phase offered all participants a second DMT dose. The primary outcome was the change in Montgomery–Åsberg Depression Rating Scale (MADRS) at 2 weeks. Secondary outcomes included response (≥50% reduction in MADRS score) and remission (MADRS ≤ 10). A total of 34 participants were randomized, 17 to placebo–active and 17 to active–active. At 2 weeks, the DMT group showed a significantly greater reduction in MADRS score than placebo (mean difference = −7.35; 95% CI = −13.62 to −1.08; *P* = 0.023). In the open-label phase, AD effects persisted up to 3 months, with no significant differences between those who received one versus two doses. Adverse events were mostly mild to moderate, commonly infusion site pain, nausea and transient anxiety. No serious adverse events occurred. A single dose of DMT with psychotherapeutic support produced a rapid, significant reduction in depressive symptoms, sustained up to 3 months. The treatment was well-tolerated and safe. ClinicalTrials.gov registration: NCT04673383.

## Abstract (web summary)

A single intravenous dose of the psychedelic dimethyltryptamine, combined with psychological support, produces rapid and lasting reductions in depressive symptoms in adults with major depressive disorder.

## Main

Major depressive disorder (MDD) is a leading cause of disability worldwide, substantially affecting individuals’ quality of life and creating a substantial public health burden^1,2^.

While selective serotonin reuptake inhibitors are commonly used as first-line treatments, many patients experience inadequate responses or unacceptable side effects^3,4^, emphasizing the need for innovative and more effective treatments. Psychedelics have emerged as a promising treatment for mood disorders^5,6^, with especially psilocybin gaining attention. Meta-analyses of randomized trials indicate that psilocybin produces moderate-to-large antidepressant (AD) effects compared to control (pooled *g* ≈ 0.66), with benefits emerging by days 8–15 and an optimal dose around 25 mg (refs. ^7–10^); these magnitudes exceed selective serotonin reuptake inhibitor meta-analytic averages (SMD ≈ *g* ≈ 0.30)^11^ and approach ketamine (intravenous (IV), SMD ≈ *g* ≈ 0.70)^12^, providing the empirical impetus to test whether similar rapid effects generalize across other classic 5-HT2A agonists, like dimethyltryptamine (N,N-DMT or DMT).

DMT is a naturally occurring tryptamine that acts largely as a serotonin 5-HT2A receptor agonist^13^. The serotonin system, and its 5-HT2AR receptors, is implicated in mood regulation, cognition and sensory processing^14^. Unlike other serotonergic psychedelics, such as oral LSD and psilocybin, IV DMT has a short half life (~5 min) that enables shorter therapeutic sessions, potentially increasing convenience and reducing costs.

Recent studies have provided initial evidence for DMT’s AD potential. A placebo-controlled study in healthy psychedelic-experienced participants found that IV DMT (20 mg) administration as a bolus injection with psychological support during dosing day significantly reduced low-level subclinical depression severity and trait anxiety scores after 1 week. These outcomes correlated with peak subjective experiences^15^. Furthermore, an open-label phase I study investigated IV DMT (0.1 mg kg^−1^ followed by 0.3 mg kg^−1^) in MDD participants and healthy controls with minimal psychological support, consisting of a 45-min preparatory session and a debriefing session with the study psychiatrist after the drug effects were weaned off. This exploratory dose-escalation study showed that DMT was safe and well-tolerated, suggesting substantial reductions in depression scores observed in MDD participants the day after dosing. The study also confirmed DMT’s rapid onset and short duration of subjective psychedelic effects when given as a bolus injection, making it a practical option for clinical use^16^. Subsequent studies in healthy volunteers have suggested that administering DMT through a slower IV infusion, as opposed to a rapid bolus, may be better tolerated and associated with fewer anxiety-related or adverse subjective effects^10,17,18^. This supports the rationale for exploring infusion-based approaches in clinical populations.

In parallel, a recent open-label trial^19^ evaluated inhaled DMT with psychological support in individuals with treatment-resistant depression (TRD), further supporting the compound’s therapeutic potential. Fourteen patients underwent a dose-escalation protocol (15 mg and 60 mg), showing rapid and sustained AD effects, with a mean Montgomery–Åsberg Depression Rating Scale (MADRS) reduction of 21.14 points by day 7. Response and remission rates reached 85.7% and 57.1%, respectively, and improvements were maintained up to 3 months. The study also confirmed the safety and tolerability of vaporized DMT, suggesting it as a promising, noninvasive alternative to IV administration in clinical settings. In this study, the psychological support element appeared to be more structured, conducted by both a psychotherapist and a psychiatrist, and focused on meaning-making, emotional or cognitive challenges from the acute phase, and promoting insights.

**Fig. 1:**
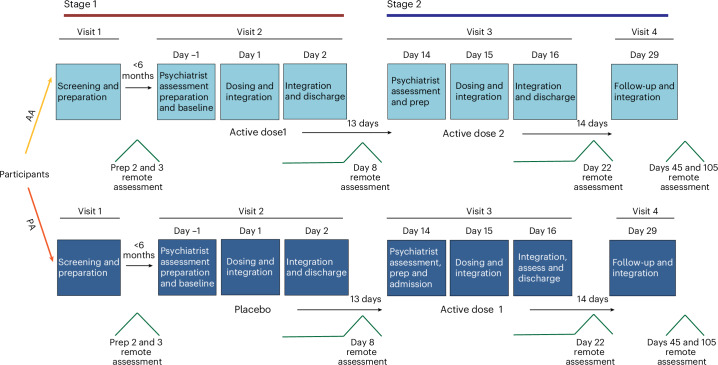
Study design and dosing schedule. Schematic overview of the two-stage phase IIa randomized, placebo-controlled trial evaluating IV DMT (SPL026) in adults with moderate-to-severe MDD. In stage 1 (double blind), participants were randomly assigned 1:1 to receive a single 21.5-mg DMT infusion (AA arm; *n* = 17) or placebo (PA arm; *n* = 17), each combined with supportive psychotherapy. In stage 2 (open label, 2 weeks later), the PA group received their first DMT dose, and the AA group received a second DMT dose. Assessments were conducted at baseline and at multiple time points up to 3 months (day 105), with an exploratory follow-up at 6 months (day 224).

Building on these findings, we conducted a two-stage phase IIa clinical trial evaluating the safety and efficacy of a 10-min IV DMT infusion in participants with moderate-to-severe MDD. The trial included a double-blind, placebo-controlled phase followed by an open-label phase (see Fig. 1 for schematics of study design and dosing schedule), with DMT (SPL026; DMT fumarate) administered in a controlled therapeutic environment supported by trained therapists. Please note that this trial is the second part of a two-part trial, the first part being a phase I dose-escalation study in healthy psychedelic-naïve participants, with results reported elsewhere^10,20^.

## Results

### Patient disposition

**Fig. 2:**
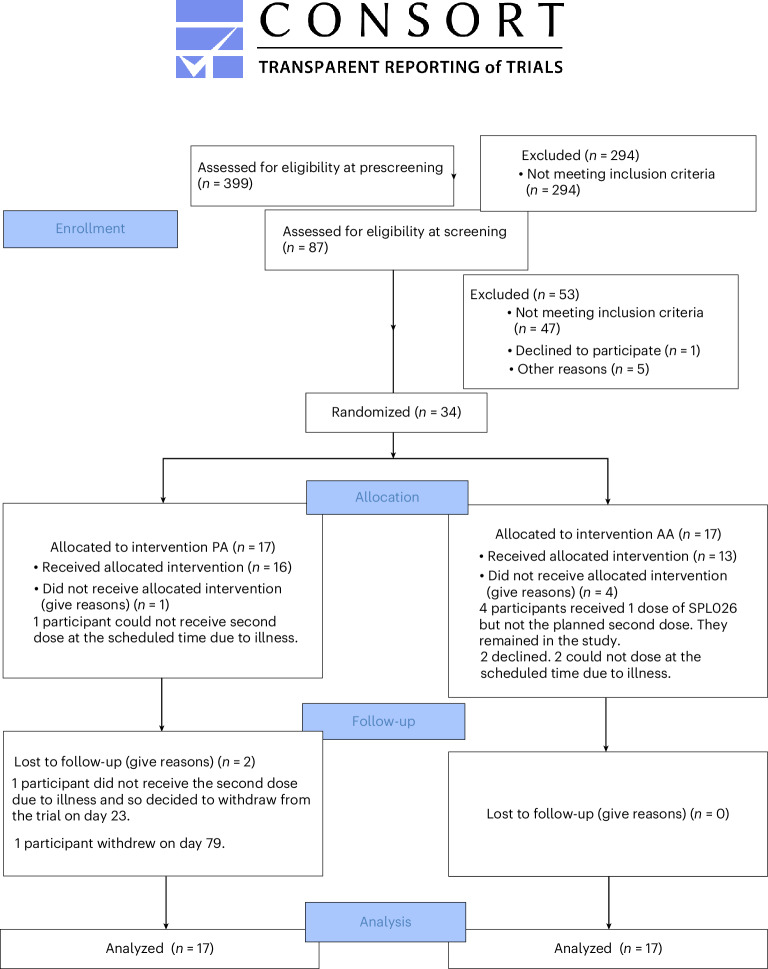
Participant flow through the trial. CONSORT diagram showing participant screening, randomization, dosing and follow-up. Of 399 individuals prescreened, 34 met inclusion criteria and were randomized (AA, *n* = 17; PA, *n* = 17). Two participants withdrew before completion. Four in the AA arm declined a second dose but completed follow-up. The diagram details attrition and numbers analyzed at each study stage. Each participant represents one independent biological replicate. CONSORT, Consolidated Standards of Reporting Trials.

Across both sites, 399 signed a prescreening consent form and were prescreened. At this stage, 312 were excluded for not meeting criteria, 105 passed prescreening and of these 87 attended a formal screening visit. Of these, 47 participants did not meet inclusion criteria, declined to participate or were excluded for other reasons (Supplementary Results (Consort diagram) and Supplementary Fig. 1). Thus, 34 participants were enrolled and underwent randomization. A total of 17 were assigned to the placebo–active (PA) group and 17 to the active–active (AA) group. Two participants withdrew from the trial. Additionally, four participants in AA group did not receive their second doses but remained in the trial (thus referred to as A−). More details can be found in Fig. 2. For explanation, see Supplementary Results (Participant withdrawals).

The study enrolled 34 participants with a mean age of 32.8 years (±9.1), of whom 29.4% were women, and the majority (88.2%) self-identified as white. Participants had experienced depression for an average of 10.5 years. At baseline, depression severity reflected similar levels of symptom intensity across groups. Eight participants were taking psychiatric medications at the time of screening, which were discontinued for at least 14 days (at least 28 days for monoamine oxidase inhibitors) before their first dosing session. A total of 11 participants reported prior psychedelic use, with all participants except two only experiencing low doses, not reporting any profound or immersive effects from the compounds. First participant was screened on 7 October 2021 and last follow-up occurred on 21 December 2022. Overall, the demographic and clinical characteristics of the groups were comparable, with additional details provided in Table 1.

**Table 1:** Demographic and baseline characteristics of the study sample, summarizing age, sex, education level and other key variables relevant to participant composition and group comparability

Characteristics		PA group (*n* = 17)	AA group (*n* = 17)	All participants (*n* = 34)
Age (years)	Mean (s.d.)	33.1 (9.73)	32.5 (8.60)	32.8 (9.05)
	Range	21–52	24–53	21–53
Sex, *n* (%)	Female	6 (35.3)	4 (23.5)	10 (29.4)
	Male	11 (64.7)	13 (76.5)	24 (70.6)
Race, *n* (%)	Asian	1 (5.9)	1 (5.9)	2 (5.9)
	Black or African American	–	1 (5.9)	1 (2.9)
	White	15 (88.2)	15 (88.2)	30 (88.2)
	Other	1 (5.9)	–	1 (2.9)
Ethnicity, *n* (%)	Hispanic or Latino	1 (5.9)	–	1 (2.9)
	Not Hispanic or Latino	16 (94.1)	17 (100.0)	33 (97.1)
Body mass index (kg m^−^^2^)	Mean (s.d.)	24.98 (3.796)	24.62 (3.855)	24.80 (3.772)
	Range	19.6–33.2	19.7–32.6	19.6–33.2
Cigarettes (daily)	*n*	1	4	5
	Range	3	2–10	2–10
Alcohol^a^ (units per week)	*n*	13	15	28
	Mean	4.4	6.4	5.5
	Range	1–14	1–18	1–18
Years with depression	Mean (s.d.)	13.0 (9.4)	7.71 (6.8)	10.4 (8.5)
	Median	10	6	7.5
	Range	2–30	1–30	1–31
Weaned off AD before dosing	*n*	4	4	8
Prior psychedelic use	*n*	5	6	11
HAM-D score day −1	Mean (s.d.)	18.65 (1.32)	19.65 (2.12)	19.15 (1.81)
	Range	17–21	17–22	17–22
MADRS score day −1	Mean (s.d.)	26.3 (6.1)	25.5 (7.2)	25.9 (6.6)
	Range	13–34	12–38	12–38

^a^Include only those who consume alcohol.

### Primary outcome

**Fig. 3:**
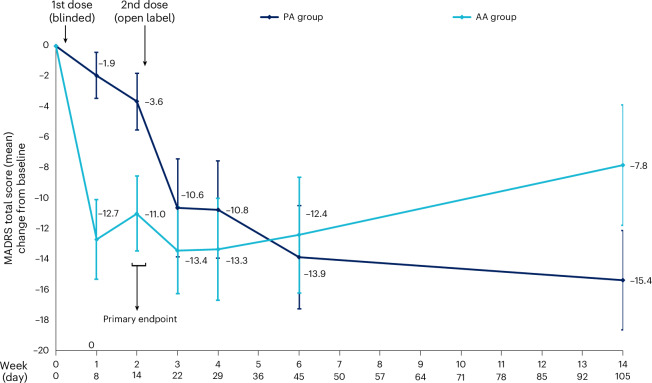
Change in depressive symptoms following DMT treatment. Mean (±s.e.m.) MADRS total scores from baseline (day −1) to 3-month follow-up (day 105). In stage 1 (double blind), participants who received DMT (AA, *n* = 17) showed a significantly greater reduction in depressive symptoms at 2 weeks compared with placebo (PA, *n* = 17; mean difference = −7.35 points; 95% CI = −13.62 to −1.08; *P* = 0.023, two-sample *t* test). During stage 2 (open label), both groups maintained improved scores up to 3 months. Error bars indicate s.e. of the mean. Analyses of the double-blinded phase used an independent samples *t* test, and analyses of the open-label phase used an MMRM.

The mean change from baseline in MADRS scores at 2 weeks after the first dose (the primary outcome) was significantly greater in participants receiving DMT fumarate compared to those receiving placebo and displayed a large effect size (mean difference = −7.35 points; 95% CI = −13.62 to −1.08; *P* = 0.023; *d* = 0.82). At 1 week (secondary outcome), the reduction in scores between the two groups was also significant (mean difference = −10.75 points; 95% CI = −16.95 to −4.55; *P* = 0.002; *d* = 1.09). In the open-label phase (stage 2), mixed model for repeated measures (MMRM) analyses revealed no significant differences in MADRS scores among participants receiving one dose of DMT fumarate (PA group) and those receiving two doses (AA group) at any follow-up time points. At trial week 3 (day 22, 1 week after the second dose), the mean difference between the PA and AA groups was −3.26 points (95% CI = −11.34 to 4.81; *P* = 0.42). Similar results were observed at week 4 (day 29 = −3.26 points; 95% CI = −11.10 to 4.59; *P* = 0.41), week 6 (day 45 = 0.16 points; 95% CI = −7.68 to 8.00; *P* = 0.96) and week 14 (day 105 = 7.03 points; 95% CI = −1.04 to 15.11; *P* = 0.08; Fig. 3, Supplementary Results (Secondary analysis—MADRS score comparison between PA and AA groups in stage) and Supplementary Table 4).

### Secondary outcomes

In participants who received two DMT doses (AA participants in stages 1 and 2), most clinical improvements in MADRS scores were observed within the initial 2 weeks after the first dose, with no significant additional effect observed after the second dose (Supplementary Results (Supplementary analysis—AA group within-participant comparison of two DMT doses) and Supplementary Table 5). Similarly, the timing of the first dose (stage 1 or stage 2) did not significantly influence the reductions in MADRS scores post-DMT (Supplementary Results (Supplementary analysis—timing of the first DMT dose: stage 1 vs stage 2) and Supplementary Table 6).

At 1 week, an MADRS response was observed in 6% PA participants and 44% AA participants (difference = 37.5 percentage points; 95% CI = 10.5 to 64.6), while remission was achieved in 13% and 44%, respectively (difference = 31.3 percentage points; 95% CI = 2.0 to 60.5). At 2 weeks, response rates were 12% for PA and 35% for AA (difference = 23.5 percentage points; 95% CI = −3.9 to 50.9), and remission rates were 12% and 29%, respectively (difference = 17.7 percentage points; 95% CI = −8.9 to 44.2). Sustained response and remission rates during the open-label phase remained stable over time, with no statistically significant differences between groups at any stage 2 time point (remission rates can be found in Supplementary Results (Supplementary analysis—MADRS remission rates) and Supplementary Table 7). Remission and response rates at 6 months for all pooled participants (one or two doses) were 40% and 44%, respectively. All MADRS score data from both groups (PA and AA) and both stages (1 and 2), including 6-month exploratory data, can be found in Table 2. Descriptive representations of Beck Depression Inventory-II (BDI-II) and State-Trait Anxiety Inventory (STAI-T) outcome data, which trended in line with the MADRS data, can be found in Supplementary Results (Secondary analysis—all BDI and STAI-T data) and Supplementary Table 3.

**Table 2:** Changes in depressive symptoms

Group	Stage	Treatment	Time	*n*	Mean (s.d.)	Change from baseline (s.d.)	Within-subject Cohen’s *d*	Response (%)
PA group (*n*_total_ = 17)	Stage 1 (blinded)	Placebo (*n*_total_ = 17)	Day −1	17	25.5 (7.25)	–	–	–
			Day 8	16	23.6 (8.29)	–1.9 (6.04)	−0.31	12.5
			Day 14	17	21.9 (10.71)	–3.6 (7.67)	−0.47	11.8
	Stage 2 (open label)	DMT (after placebo; *n*_total _= 16)	Day 22	14	14.4 (11.72)	–10.6 (12.03)	−0.88	42.9
			Day 29	16	14.4 (10.93)	–10.8 (12.77)	−0.85	43.8
			Day 45	16	11.9 (9.94)	–13.3 (12.93)	−1.02	50.0
			Day 105	14	9.6 (8.05)	–15.4 (12.21)	−1.26	50.0
			Day 224	12	14.4 (9.82)	–8.4 (12.45)	−0.67	50.0
AA group (*n*_total_ = 17)	Stage 1 (blinded)	DMT (*n*_total_ = 17)	Day −1	17	26.3 (6.14)	–	–	–
			Day 8	16	13.3 (10.36)	–12.7 (10.38)	−1.22	43.8
			Day 14	17	15.3 (10.20)	–11.0 (10.11)	−1.35	35.3
	Stage 2 (open label)	DMT (after active; *n*_total_ = 13^a^)	Day 22	12	11.1 (8.53)	–13.4 (9.89)	−1.15	58.3
			Day 29	12	11.2 (9.49)	–13.3 (11.57)	−0.94	58.3
			Day 45	12	12.1 (10.29)	–12.4 (13.13)	−0.94	50.0
			Day 105	12	16.7 (12.26)	–7.8 (13.63)	−0.59	41.7
			Day 224	9	16.0 (7.79)	–7.8 (12.02)	−0.64	33.3
All participants (*n*_total_ = 34)	Stage 2 (open label)	DMT (one or two doses (*n*_total _= 33)	Day 105	30	13.7 (11.15)	−11.8 (13.16)	–	46.7
			Day 224	25	15.2 (9.28)	−9.2 (12.0)	–	44.0

MADRS outcome data at all time points and in both stages, including within-subject Cohen’s *d* (relative to day −1) and response rates (response defined as ≥50% change from baseline). *n*_total_, total number of participants; *n*, number of participants with data. ^a^AA data are without the 4 individuals (A−) who did not receive their second DMT dose.

### Safety

The treatment was overall well-tolerated in male and female participants with MDD who received one or two IV doses of DMT fumarate. Treatment-emergent adverse events (TEAEs) occurred in the majority of participants, with 25 individuals (73.5%) reporting 47 TEAEs deemed possibly related to treatment by the investigator. The severity of these TEAEs was largely mild, observed in 15 participants, while 10 participants experienced moderate TEAEs. They mostly resolved over the dosing visit. The incidence of TEAEs was higher after the first dose of DMT fumarate (64.7% in stage 1 and 62.5% in stage 2) compared to placebo (23.5%). Interestingly, a second dose of DMT fumarate in stage 2 in the AA group was associated with a lower incidence of treatment-related TEAEs (23.1%), a proportion similar to that seen in the placebo group (Table 3).

**Table 3:** TEAEs deemed related to treatment by investigator

System organ class Preferred term	PA group		AA group		All participants (*n*_total_ = 34)
	Stage 1	Stage 2	Stage 1	Stage 2	
	Placebo (*n*_total_ = 17)	DMT after placebo (*n*_total_ = 16)	DMT (*n*_total_ = 17)	DMT after active (*n*_total_ = 13)	
	*n* (%) (number of events)				
Total	4 (23.5)	10 (62.5)	11 (64.7)	3 (23.1)	25 (73.5)
General disorders and administration site conditions	3 (17.6)	6 (37.5)	7 (41.2)	1 (7.7)	16 (47.1)
Infusion site pain	3 (17.6) (3)	6 (37.5) (6)	6 (35.3) (6)	1 (7.7) (1)	15 (44.1) (16)
Catheter site-related reaction	–	–	1 (5.9) (1)	–	1 (2.9) (1)
Psychiatric disorders	1 (5.9)	6 (37.5)	3 (17.6)	1 (7.7)	10 (29.4)
Anxiety	–	3 (18.8) (3)	2 (11.8) (2)	–	5 (14.7) (5)
Insomnia	1 (5.9) (1)	1 (6.3) (1)	–	–	2 (5.9) (2)
Restlessness	–	1 (6.3) (1)	1 (5.9) (1)	–	2 (5.9) (2)
Depression	–	1 (6.3) (1)	–	–	1 (2.9) (1)
Emotional distress	–	1 (6.3) (1)	–	–	1 (2.9) (1)
Middle insomnia	–	–	–	1 (7.7) (1)	1 (2.9) (1)
Pseudohallucination	–	–	1 (5.9) (1)	–	1 (2.9) (1)
Gastrointestinal disorders	–	3 (18.8)	3 (17.6)	**–**	6 (17.6)
Nausea	–	3 (18.8) (3)	3 (17.6) (3)	–	6 (17.6) (6)
Nervous system disorders	–	1 (6.3)	3 (17.6)	–	4 (11.8)
Headache	–	1 (6.3) (1)	1 (5.9) (1)	–	2 (5.9) (2)
Disturbance in attention	–	–	1 (5.9) (1)	–	1 (2.9) (1)
Paresthesia	–	–	1 (5.9) (1)	–	1 (2.9) (1)
Musculoskeletal and connective tissue disorders	–	1 (6.3)	1 (5.9)	–	2 (5.9)
Muscle tightness	–	1 (6.3) (1)	–	–	1 (2.9) (1)
Pain in extremity	–	–	1 (5.9) (1)	–	1 (2.9) (1)
Vascular disorders	–	**–**	2 (11.8)	**–**	2 (5.9)
Hypertension	–	–	1 (5.9) (1)	–	1 (2.9) (1)
Pallor	–	–	1 (5.9) (1)	–	1 (2.9) (1)
Ear and labyrinth disorders	**–**	**–**	**–**	1 (7.7)	1 (2.9)
Tinnitus	–	–	–	1 (7.7) (1)	1 (2.9) (1)
Eye disorders	**–**	**–**	1 (5.9)	**–**	1 (2.9)
Visual snow syndrome	–	–	1 (5.9) (1)	–	1 (2.9) (1)
Skin and subcutaneous tissue disorders	**–**	1 (6.3)	**–**	**–**	1 (2.9)
Night sweats	–	1 (6.3) (1)	–	–	1 (2.9) (1)

TEAEs occurring from randomization (dosing) on day 1 to day 14 inclusive are included in stage 1. TEAEs occurring from dosing on day 15 until the end of the study are included in stage 2. TEAEs that occurred on the day of, but before dosing in stage 2 (day 15), are not included in the table. Participants with ≥1 TEAE are counted only once per system organ class and preferred term. *n*_total_, total number of participants; *n*, number of participants with a TEAE.

The most frequent treatment-related TEAEs reported after DMT were infusion site pain (3 after placebo and 13 after DMT), nausea (0 after placebo, 6 after DMT), anxiety (0 after placebo, 6 after DMT), headache (0 after placebo, 2 after DMT), insomnia (1 after placebo, 1 after DMT) and restlessness (0 after placebo, 2 after DMT). Notably, there were no serious adverse events related to treatment, no deaths or TEAEs that necessitated withdrawal from the study, dose discontinuation or adjustment. A table listing all TEAEs (including those deemed not related to treatment) can be found in Supplementary Results (Safety results) and Supplementary Table 14.

Clinical evaluations, including physical examinations, laboratory analyses and electrocardiograms (ECGs), revealed no clinically significant abnormalities throughout the trial. However, transient and discrete increases in blood pressure and heart rate were observed immediately after DMT administration (Supplementary Results (Safety results)).

All participants tolerated the dosing sessions well, except for one individual who expressed regret after their second dose (Supplementary Results (Safety results)). Throughout the study, no meaningful or concerning changes in suicidal ideation were recorded based on the Beck Scale for Suicidal Ideation (Supplementary Results (Safety results) and Supplementary Table 18).

### Exploratory outcomes

AD effects 2 weeks after participants’ first active DMT dose were observed to be moderated by their ‘Mystical Experience’ Questionnaire (MEQ) scores, as well as Ego Dissolution Inventory, feelings of Unity (11 Dimensions Altered States of Consciousness questionnaire) and subject-led visual analog scales (Individual-Rated Visual Analog Scales) general intensity scores (Supplementary Results (Supplementary materials—acute measures and moderation effects of Mystical Experience (MEQ) scores and related measures of the acute psychedelic experience)). We also explored whether AD washout, prior psychedelic use or psychedelic predictor scale subscales (set, setting, rapport, intention) moderated treatment outcomes. None of these factors showed a significant effect on AD response at 2 weeks. Full results and statistical tables are provided in Supplementary Results (Supplementary analyses on possible effects of antidepressant washout, prior experience with psychedelics and preparation (set, setting, therapeutic rapport and intention) on antidepressant effects). Given the small subgroup sizes and limited power, these findings should be interpreted with caution.

## Discussion

This phase IIa trial evaluated the efficacy, safety and tolerability of one or two 21.5-mg IV 10-min infusions of DMT fumarate with supportive therapy in participants with moderate-to-severe MDD. Participants were randomized to receive either DMT or placebo in a blinded phase, followed 2 weeks later by an open-label phase where all participants could have received active treatment. The primary endpoint, change in MADRS scores from baseline to 2 weeks, demonstrated a significant improvement after DMT treatment compared with placebo, confirming DMT’s superior AD efficacy to placebo in the blinded phase of the trial. Thus, the trial’s main endpoint was met.

Secondary analyses showed a rapid reduction in MADRS scores by 1 week, and concomitant improvements in response and remission rates generally aligned with the primary outcome. In the open-label phase, the AD effects were durable over 12 weeks (and up to 6 months in some participants), with no notable differences between single-dose and two-dose regimens, suggesting that one dose may suffice for sustained benefits. Nonetheless, at the 3-month follow-up, the PA group exhibited a numerically greater reduction in depressive symptoms compared to the AA group (~7.6 points on the MADRS), a difference similar in magnitude to the significant treatment effect observed at 2 weeks (7.35 points, *P* = 0.023). However, this longer-term difference did not reach statistical significance (*P* = 0.08). This may be due in part to reduced statistical power in part B, where participant dropouts exceeded 10% (9 of 34 participants), limiting the ability to detect between-group differences at later time points. One possible mechanistic explanation for the greater sustained response in the PA group is the longer preparatory phase before dosing. Extended therapist–participant interaction may have facilitated a stronger therapeutic alliance, which is increasingly recognized as a key contributor to outcomes in psychedelic-assisted therapy^21^. While this remains speculative, the trend observed here highlights the need for future studies that both minimize attrition and directly measure therapeutic alliance to better understand its role in shaping long-term treatment effects. The absence of formal measures of therapeutic rapport or alliance in the present study also limits our ability to evaluate this hypothesis directly and should be addressed in future research.

DMT was well-tolerated, with no serious adverse events or instances of suicidal ideation or suicidal behavior deemed related to the study drug reported. Most adverse events were mild or moderate, and the most common was infusion site pain. A second dose was associated with fewer adverse events than the first, and most resolved during the dosing visit. No substantial safety concerns were identified in laboratory findings, ECG or vital signs, supporting the overall tolerability of the treatment. Unlike psilocybin, which has been associated with postdose headaches in 40–50% participants in previous studies^22^, DMT did not appear to reliably induce headaches in this study.

The between-group effect size for the reduction in depressive symptoms at 2 weeks in the present study (*d* = 0.82) is comparable to the effect size observed in a previous trial of psilocybin (25 mg versus niacin) with psychological support in MDD participants (*d* = 0.90)^23^ and greater than the 3-week between-group effect size of psilocybin (25 mg versus 1 mg) in TRD (*d* = 0.55)^24^.

Similar to other psychedelic compounds, DMT’s AD effects may be linked to its ability to promote neuroplasticity^25^, its transient—but substantial—impact in brain function in associative regions^15,26,27^ or (relatedly) to its subjective effects^15^, with the present results suggesting that reductions in depression were moderated by the intensity of mystical-type experiences induced during treatment (Supplementary Results (Supplementary materials—acute measures and moderation effects of Mystical Experience (MEQ) scores and related measures of the acute psychedelic experience)). Due to its shorter duration of action, IV DMT treatment could offer a more time-efficient, practical and scalable option for psychedelic-based treatments. A recent open-label phase IIa trial using inhaled DMT (escalating 15 mg and then 60 mg) in TRD also reported rapid and sustained AD effects over a 3-month period, with large within-subject effect sizes (for example, *g* = 2.20 at day 7, *g* = 1.33 at 3 months)^19^. While methodological differences make direct comparisons difficult, variations in dose, route of administration and study design, including the use of a placebo control in the present study, may partly account for the somewhat smaller between-group effect size observed at 2 weeks (*d* = 0.82). Together, these studies highlight the importance of further controlled research to clarify the role of dosing, setting and delivery methods in shaping clinical outcomes.

This study has several limitations. The sample was not ethnically diverse, which may limit the generalizability of the findings. Participants with a history of serious suicide attempts were excluded. Additionally, we did not assess blinding integrity or participant expectancy, which may have influenced outcomes, particularly given the distinct subjective effects of DMT. If blinding is compromised, expectations about improvement may influence outcomes, making it harder to isolate pharmacological effects from psychological or contextual ones (although see ref. ^28^ for evidence suggesting that expectancy may not fully account for psychedelic treatment effects). We explored whether AD washout or prior psychedelic experience influenced AD response at 2 weeks. Neither factor showed an effect, although the small subgroup sizes limit the strength of these conclusions (Supplementary Results (Supplementary analyses on possible effects of antidepressant washout, prior experience with psychedelics and preparation (set, setting, therapeutic rapport and intention) on antidepressant effects)). Moreover, the blinded first dose and the open label nature of the second dose limit the comparisons that can be made between one and two doses. The results of this second phase of the study must thus be interpreted as hypothesis-generating. DMT was administered alongside a time-limited, relational psychotherapeutic framework focused on psychological flexibility. Given the hypothesis of synergistic effects between psychedelic compounds and therapeutic support^29^, it remains unclear whether similar results would be achieved with minimal psychological support. Formal measures on the quality of psychological integration were also not collected, limiting our capacity to draw conclusions on the influence of the psychological intervention on clinical outcomes.

In this phase IIa trial, a 21.5-mg dose of DMT administered as a 10-min IV infusion with psychological support resulted in a significantly greater reduction in depression at 2 weeks compared with placebo in participants with moderate-to-severe MDD. The treatment demonstrated a rapid onset of effect, with improvements observed as early as the first assessment time point of 1 week, and AD effects were sustained during the 12-week open-label phase. Longer and larger trials, including comparisons with existing treatments, are needed to further evaluate the efficacy, safety and cost-effectiveness of DMT in the treatment of MDD.

## Methods

### Trial oversight

This was a phase IIa double-blind, placebo-controlled, randomized clinical trial. The sponsor, Small Pharma (now Cybin UK), designed and supported the trial and provided a proprietary pharmaceutical-grade formulation of DMT fumarate (SPL026), which was analyzed for stability and purity. The trial evaluated the efficacy and safety of DMT, administered as a single or double IV dose, to participants with MDD. The trial was conducted at Hammersmith Medicines Research (HMR), MAC Clinical Research (MAC) and Imperial College London Hammersmith Campus under their respective standard operating procedures.

HMR supervised the trial’s execution, and independent site raters conducted blinded assessments of participants using validated tools, ensuring raters were unaware of trial-group assignments. Data management and analysis were performed by HMR, reviewed by the sponsor and supplemented with post hoc statistical analyses conducted by the sponsor. Small Pharma, as Sponsor, had full oversight of the clinical trial. Data were collected via an electronic case report form using the Medrio EDC system (latest version at the time of use, 2020–2022).

The trial adhered to International Council for Harmonization Good Clinical Practice guidelines and the ethical principles of the Declaration of Helsinki. The trial protocol was reviewed and approved by the UK Medicines and Healthcare products Regulatory Agency and the London–Brent Research Ethics Committee. All participants provided written informed consent. The trial is registered on ClinicalTrials.gov (NCT04673383) and ISRCTN (ISRCTN63465876), where the clinical study report synopsis is available.

### Participants

Adults aged 18 years or over with a diagnosis of moderate-to-severe MDD and a history of at least two prior unsuccessful treatment attempts (pharmacological or/and psychotherapeutic) were recruited formally through databases held by the clinical trial sites, informally through social media, and through other sources, which directed participants to a recruitment website. The main exclusion criteria were an immediate family or personal history of psychosis, medically significant health conditions that make a person unsuitable to participate in the trial (as assessed by a physician), a history of serious suicide attempts, a positive pregnancy test, the use of serotonergic psychedelic drugs in the previous 6 months or the suspected or known presence of a pre-existing psychiatric condition (for example, borderline personality disorder) that could jeopardize rapport between the participant and their two trial therapists in light of the protocol-defined limits of available therapist support. Additional details about the trial exclusion criteria are provided in Supplementary Methods (Inclusion and exclusion criteria).

Volunteers initiated contact by emailing the recruitment coordinator after hearing about the trial. Most of the recruited participants referred themselves and some were recruited through patient databases. Candidates were sent a participant information sheet and invited to a telephone screening. Initial assessments of eligibility and suitability were performed by means of a video call. Confirmation of a diagnosis of depression and medical history were obtained from the participant’s general physician. Eligible participants then underwent face-to-face physical and mental health assessments (including the Mini-International Neuropsychiatric Interview) with a trial physician, psychiatrist and therapist (visit 1). Assessment with the 17-item Hamilton Depression Scale (HAM-D-17) was done by the site psychiatrist; a score of at least 17 (indicating moderate-to-severe MDD) on a scale that ranges from 0 to 52, with higher scores indicating greater depression, was required for trial enrollment. Any participants taking AD medication at screening discontinued the use before starting the trial (with the support of a study psychiatrist), with full discontinuation occurring at least 2 weeks before starting a trial medication.

Participants were financially compensated for their time.

The tables use the term sex rather than gender, but this was determined by self-reporting and may reflect gender identity rather than sex assigned at birth. Sex-based/gender-based analyses have not been performed due to overall low sample size. The race/ethnicity categories used followed the FDA’s recommendations which are as follows: American Indian or Alaska Native; Asian; Black or African American; Native Hawaiian or other Pacific Islander; and White. Participants were allowed to self-report and could also designate themselves as multiracial. Ethnicity was separated from race, with the categories—Hispanic/Latino or not Hispanic/Latino. These categories were not used for any inferences or analyses.

### Trial design

The sponsor-initiated trial reported here constitutes part B of a larger study investigating the safety, tolerability and efficacy of DMT fumarate in healthy participants (part A) and participants with MDD (part B). Findings from part A, conducted in healthy participants, have been published previously^10,20^ and the remainder of this manuscript refers to part B only. Enrolled participants with MDD followed a two-stage, double-blind, randomized, placebo-controlled design, with a planned enrollment of 28 to 36 participants (34 were randomized).

Participants received up to two single IV doses of DMT fumarate or placebo accompanied by a time-limited, relational psychotherapeutic framework focused on psychological flexibility (Supplementary Methods (Therapeutic support)). In stage 1, participant numbers were allocated to blinded treatments (active or placebo) according to a 1:1 randomization schedule prepared by an independent HMR statistician, using SAS statistical analysis software. Two weeks later, in stage 2, participants received a second, open label, dose of DMT, either as their first exposure for those who received placebo in stage 1, defined as PA arm (PA group) or as a second dose for those who received DMT in stage 1, defined as AA arm (AA group). Participants who declined a second dose or were deemed unsuitable for further dosing by the study psychiatrist proceeded to follow-up assessments after their first dose (defined as A- arm). Due to the noticeable psychedelic effects of DMT fumarate, it was likely that both participants and investigators could determine whether a participant had received the active drug or placebo. Despite this, stage 1 of the study adhered to double-blind principles, while stage 2 was conducted as an open-label phase. More information on blinding is provided in the Supplementary Methods (Blinding).

The dosing regimen was based on data from part A and selected for safety and tolerability. The dose administered was 21.5-mg DMT fumarate, infused intravenously over 10 min in two phases—6 mg over the first 5 min, followed by 15.5 mg over the subsequent 5 min (see refs. ^10,20^ for details). Details of the screening process can be found in Supplementary Methods (Trial visits).

Dosing took place in a controlled inpatient setting, with participants admitted to the ward 1 day prior and discharged the following morning. Preparation involved a 90-min session with two therapists the day before dosing, including orientation to the setting, a guided visualization to promote relaxation and interpersonal safety, and discussion of participant expectations. On dosing day, an additional brief session supported emotional readiness and anticipatory anxiety. Participants received IV DMT in a nonclinical, softly lit room designed to promote calm and containment. They wore eyeshades and noise-canceling headphones and listened to a specially curated, nonverbal ambient music playlist produced by M. Cooper. The music was tailored to follow the experiential trajectory of the DMT session, expansive and immersive during the peak, then gently grounding toward the end. Two therapists were present throughout the session to provide silent, attentive support, while a study psychiatrist remained on site.

Integration was delivered using a relational and participant-led framework developed from previous psilocybin trials. This approach encourages open exploration of emotional, embodied and transpersonal elements of the experience, without prematurely fixing meaning. Integration sessions were conducted in person on day 1 and day 2 postdose, and again on day 15, with therapists helping participants reflect on the psychedelic material and explore how insights could be applied to daily life. Additional follow-up check-ins supported ongoing integration and monitored well-being. Full procedural details for trial visits and the therapeutic framework are provided in the Supplementary Methods (Trial visits and Therapeutic support).

Remote follow-up assessments were conducted 1-week postdose, an in-person visit at day 29 and remote visits 1 month, and 3 months after the final dose. MADRS was assessed (using the SIGMA guide) by a trained independent site rater who was not present during dosing. An overview of the trial is shown in Fig. 1.

In addition to the main trial, an exploratory out-of-study 6-month follow-up visit was performed to monitor long-term outcomes (Supplementary Methods (Trial visits)).

### Power calculations

The sample size calculation was based on a two-sided, two-sample *t* test with equal variance at a significance level of 0.05 and a 1:1 allocation ratio, using data from ref. ^30^. A sample size of 28 to 36 participants provided 80% to 90% power to detect a 12.5-point mean difference in MADRS score change from baseline.

### Efficacy outcomes

The primary outcome of this trial was the change from baseline in the MADRS^31^ score at 2 weeks (day 14; ±2 days) after the first dose. Secondary efficacy measures included MADRS scores assessed at 1 week (day 8; ±1 day) after the first dose and at 1, 2, 4 and 12 weeks after the second dose (day 22; ±1 day, 29; ±2 days, 45; ±2 days and 105; ±5 days). Additional psychometric tools included the BDI-II^32^ and the Spielberger’s STAI-T^33^. These instruments were used to evaluate broader aspects of mood and anxiety that complement the primary depression-related outcome. Participants who did not receive a second dose but remained in the study completed the same follow-up assessments as other participants. The BDI-II and STAI-T were administered in paper form for self-assessment. MADRS was completed on a paper form by an independent site rater after the SIGMA guide^34^. Instructions were provided at the top of the first page of each questionnaire. The MEQ^35^, as well as other subjective measures like Ego Dissolution Inventory^36^, Emotional Breakthrough Inventory^37^, 11 Dimensions Altered States of Consciousness^38^, Psychological Insights Scale^39^, Challenging Experience Questionnaire^40^ and Individual-Rated Visual Analog Scales, were used to measure the subjective effects of DMT. These subjective scores were also examined to see if they could predict subsequent AD effects. We also examined whether elements of psychedelic preparation (therapeutic rapport, intentions, set and setting) influenced AD response at 2 weeks using the psychedelic predictor scale^41^. Exploratory analyses also assessed for possible influence of AD washout and prior psychedelic use on AD effects at 2 weeks. A completed list of the measures collected in this trial, including the ones not reported here, can be found in Supplementary Methods (Complete list of collected measures) and Supplementary Table 1.

### Safety and tolerability

Adverse events were recorded throughout the study and categorized by severity (mild, moderate, severe) and relationship to treatment (possibly or unlikely related). TEAEs were those occurring postrandomization—from dosing on day 1 to day 14 (stage 1) or from day 15 to day 105 (stage 2). Day 15 TEAEs occurring before dosing on day 15 were excluded from summary tables. Serious adverse events were defined per regulatory guidelines and managed by the sponsor. Additional details on adverse event collection are provided in the Supplementary Methods (Additional adverse event collection methods). Safety monitoring included blood pressure and heart rate (predose and at 12, 60 and 240 min after dosing), ECGs (predose and at 180 min after dosing) and lab tests. Tolerability was assessed postdose by asking if participants regretted the experience. Injection site assessments recorded pain, tenderness, erythema or induration.

### Statistical analyses

The primary endpoint was the change in MADRS score between the AA and PA groups from baseline to week 2, with the week 1 change as a secondary outcome, assessed using two-sample *t* tests during the blinded phase (stage 1). Secondary endpoints, including sustained effects of DMT and the potential benefit of an additional dose, were analyzed using an MMRM. The first MMRM analysis compared MADRS score changes at 1 week (day 22), 2 weeks (day 29), 1 month (day 45) and 3 months (day 105) during the open-label phase (stage 2) between participants who received a single DMT dose (PA group) and those who received two doses (AA group). Additional MMRM analyses explored within-participant changes in MADRS scores after each dose in the AA group and assessed whether the timing of the first DMT dose influenced clinical outcomes (Supplementary Results (‘Secondary analysis—MADRS score comparison between PA and AA groups in stage’ to ‘Supplementary analysis—MADRS remission rates’)). Response and remission rates, defined as >50% reduction in MADRS score and an MADRS score ≤10, respectively, were analyzed using logistic regression adjusted for baseline scores. Changes in BDI-II and STAI-T scores and MADRS scores at the 6-month follow-up (day 224) were summarized descriptively. Moderation effects of the acute psychedelic experience, measured by MEQ scores, on depression severity were explored (Supplementary Results (Supplementary materials—acute measures and moderation effects of Mystical Experience (MEQ) scores and related measures of the acute psychedelic experience) and Supplementary Fig. 2). In response to reviewer feedback, a worst-case scenario sensitivity analysis was conducted to evaluate robustness against potential nonrandom missingness of data in the open-label phase. These results are presented in Supplementary Results (Imputation analyses for handling missing data) and confirmed the results of the main analyses. All analyses, excluding the moderation effects, were done after the statistical analysis plan had been signed and after the database had been locked. The statistical analyses were done by an HMR statistician, using SAS (version 9.4; moderation effects were analyzed by Imperial College using R, the latest version at the time of analysis).

### Reporting summary

Further information on research design is available in the Nature Portfolio Reporting Summary linked to this article.

## Online content

Any methods, additional references, Nature Portfolio reporting summaries, source data, extended data, supplementary information, acknowledgements, peer review information; details of author contributions and competing interests; and statements of data and code availability are available at 10.1038/s41591-025-04154-z.

## Supplementary information

Supplementary Information Supplementary Methods, Supplementary Results, Supplementary Tables 1–18 and Supplementary Figs. 1–7.

Reporting Summary

## Data Availability

The clinical trial data underlying this article, including protocol and statistical analysis plan, are not publicly available due to privacy concerns and restrictions from the trial sponsor (Cybin IRL). In line with ICMJE guidelines, de-identified participant data will be made available upon reasonable request to the sponsor for the purposes of verifying the results within 1 month of execution of a data-sharing agreement. Access will be granted to qualified researchers following review and subject to a data-sharing agreement to ensure participant confidentiality and compliance with regulatory requirements.
