# Supplementary material for: A short-acting psychedelic intervention for major depressive disorder: a phase IIa randomized placebo-controlled trial
Source: Nat Med. 2026 Feb 16;32(2):591–8. doi: 10.1038/s41591-025-04154-z (PMC12920121; doi:10.1038/s41591-025-04154-z)
Supplement: Supplementary file 1 — Supplementary Methods, Supplementary Results, Supplementary Tables 1–18 and Supplementary Figs. 1–7. [file 41591_2025_4154_MOESM1_ESM.pdf]

nature medicine

Supplementary information https://doi.org/10.1038/s41591-025-04154-z

A short-acting psychedelic intervention for major depressive disorder: a phase IIa randomized placebo-controlled trial

In the format provided by the authors and unedited

1. Table of Contents

1. Table of Contents *1*

1.1. Tables and Figures 2

2. Supplementary Methods *4*

2.1. Inclusion and Exclusion Criteria 4

2.2. Dietary and Lifestyle Restrictions 5

2.2.1. Dietary Restrictions 5
2.2.2. Substance Restrictions 5
2.2.3. Activity Restrictions 5
2.2.4. Contraceptive Requirements 5

2.3. Blinding 6

2.4. Trial Compliance 7

2.5. Trial Visits 7

2.5.1. Screening 7
2.5.2. Main Trial 7
2.5.3. Optional Exploratory 6-month follow-up 8

2.6. SPL026 (DMT fumarate) Drug Product 8

2.7. Therapeutic Support 9

2.8. Complete list of collected measures 11

2.9. Additional Adverse Event Collection Methods 13

2.10. Sample Size - Rationale 13

2.11. Handling of Missing Data 14

3. Supplementary Results *14*

3.1. Consort Diagram 14

3.2. Previous and concomitant medication 15

3.2.1. Overview 15
3.2.2. Previous Medications 16
3.2.3. Concomitant Medications During the Trial 16
3.2.4. Adverse Event-Driven Medications 17

3.3. Participant Withdrawals 17

3.3.1. Withdrawals during the trial 17
3.3.2. Subjects not receiving a second dose 17
3.3.3. Follow-Up for Withdrawn Participants 17
3.3.4. Immediate Discontinuation of Treatment 17

3.4. Protocol Deviations 18

3.4.1. Major Protocol Deviations 18
3.4.2. Minor Protocol Deviations 18

3.5. Secondary Analysis – all BDI and STAI-T data 19

3.6. Secondary Analysis - MADRS Score Comparison Between PA and AA Groups in Stage 2 .19

3.7. Supplementary Analysis - AA Group Within-Participant Comparison of Two DMT Doses20

3.8. Supplementary Analysis - Timing of the First DMT Dose: Stage 1 vs Stage 2 21

3.9. Supplementary Analysis - MADRS Remission Rates 21

3.10. Supplementary Materials – Acute Measures and moderation effects of Mystical Experience (MEQ) Scores and related measures of the acute psychedelic experience...............22

3.10.1. Acute measures 22
3.10.2. Mystical Experience Questionnaire (MEQ) - Moderation Analyses 25
3.10.3. Other measures of the acute subjective psychedelic experience - Moderation and Correlation Analyses 26

3.11. Supplementary analyses on possible effects of antidepressant washout, prior experience with psychedelics and preparation (set, setting, therapeutic rapport and intention) on antidepressant effects...28

3.11.1. Effects of antidepressant washout on treatment outcomes.. 28
3.11.2. Effects of prior psychedelic use on treatment outcomes 28
3.11.3. Effects of set, setting, rapport, and intention on treatment outcomes.................................... 29
3.11.4. Adjustment for baseline depression duration... 30

3.12. Imputation analyses for handling missing data...30

3.13. Safety Results...31

3.13.1. Adverse Events and Serious Adverse Events.. 31
3.13.2. Vital Signs 35
3.13.3. 12-Lead Electrocardiogram 40
3.13.4. Injection Site Reactions 40
3.13.5. Subjective Tolerability 40
3.13.6. Clinical Chemistry and Coagulation 40
3.13.7. Suicidal Ideation 41

4. Supplementary References.. 43

1.1. Tables and Figures

Table S1: All Scales and questionnaires 13
Table S2: Sample size requirements 14
Table S3: BDI-II and STAI-T Outcome data at all time points and in both stages 19
Table S4: Comparison of MADRS score changes 20
Table S5: Within-participant comparison of MADRS score changes 20
Table S6: Comparison of MADRS score changes at 1 and 2 weeks between participants.. 21
Table S7: MADRS Remission Rates Over Time... 22
Table S8: MEQ-30 scale scores 22
Table S9: Altered States of Consciousness Questionnaire (11D-ASC) factor and total scores across placebo and DMT treatment conditions... 24
Table S10: Effects of antidepressant washout ANCOVA 28
Table S11: Effects of prior psychedelic use ANCOVA... 29
Table S12: Effects of baseline depression ANCOVA 30
Table S13: Imputation analysis.. 31
Table S14: All Treatment-emergent adverse events 34

Table S15: Summary of Vital Signs – Systolic Blood Pressure 37
Table S16: Summary of Vital Signs – Diastolic Blood Pressure 38
Table S17: Summary of Vital Signs – Heart Rate 39
Table S18: Summary of Beck Scale for Suicidal Ideation (BSS) Full 42

Figure S1: Dosing room 10
Figure S2: CONSORT flow diagram 15
Figure S3: Mystical Experience Questionnaire (MEQ-30) total scores across placebo and DMT conditions (Stage 1 and Stage 2) 23
Figure S4: Altered States of Consciousness Questionnaire (11D-ASC) factor scores for placebo and DMT treatment (Stage 1 and Stage 2) 25
Figure S5: Line plot illustrating the moderating effect of Mystical Experience 26
Figure S6: Exploratory correlational analyses between subjective measures of the acute psychedelic experience and changes in depressive symptoms at 2 weeks 27
Figure S7: Exploratory correlational analyses between different elements of psychedelic preparation and changes in depressive symptoms at 2 weeks... 29

2. Supplementary Methods

2.1. Inclusion and Exclusion Criteria

Inclusion Criteria

Participants were eligible for the trial if they met the following criteria:

1. Adults (≥18 years) with a diagnosis of moderate-to-severe major depressive disorder (MDD) as defined by the DSM-5 and a HAM-D score of ≥17.
2. Failure to achieve adequate response to at least two standard MDD treatment options.
3. Willingness to discontinue antidepressant medication (e.g., SSRIs) before and during the trial, with adequate washout periods.
4. No use of psychedelic drugs in the six months before dosing.
5. Registration with a GP or mental healthcare professional in the UK to confirm diagnosis and previous treatment.
6. Body mass index (BMI) between 18.0 and 33.9 kg/m².
7. Sufficient intelligence and communication skills to understand the trial and comply with its requirements.
8. Provision of written informed consent after discussing the trial with the investigator.
9. Agreement to adhere to contraceptive requirements (see 2.2.4).
10. Agreement not to donate blood or blood products during the study and for up to three months after dosing.
11. Willingness to refrain from using psychedelic drugs (other than the study drug) during the trial and for three months afterward.
12. Ability to be contacted via email or telephone/video call and access to online communication.
13. Consent to have data entered into The Overvolunteering Prevention System (TOPS).
14. Suitable veins for cannulation for infusion and blood sampling.

Exclusion Criteria

Participants were excluded if they met any of the following criteria:

1. Diagnosis of a psychotic disorder or first-degree family history of psychosis.
2. Significant history of mania or psychiatric conditions incompatible with safe DMT exposure (e.g., borderline personality disorder).
3. Current use of more than 20 cigarettes per day or inability to be nicotine-free during dosing periods.
4. Current alcohol intake exceeding 21 units per week.
5. Pregnant or lactating women or women of childbearing potential not using an acceptable method of contraception.
6. Clinically relevant abnormal medical history, physical findings, or laboratory results that could interfere with trial objectives or safety.
7. Acute or chronic illnesses, including neurological, endocrine, hepatic, or cardiovascular conditions, or significant medical conditions such as arrhythmia or hypertension.
8. History of serious suicide attempts requiring hospitalisation.
9. Severe adverse reactions to any drug or sensitivity to serotonergic psychedelic drugs.

10. Use of prohibited prescription or over-the-counter medications or supplements within 28 days before dosing (e.g., St John's Wort). Antidepressant medications had to be discontinued for at least 14 days (28 days for MAOIs).
11. Participation in another clinical trial within three months before admission.
12. Drug or alcohol abuse or dependence or positive urine drug test results.
13. Blood pressure, heart rate, or QTcF values outside acceptable ranges at screening (e.g., QTcF >450 ms for men or >470 ms for women).
14. Positive test results for hepatitis B, hepatitis C, or HIV.
15. Blood loss exceeding 400 mL within three months before the trial.
16. Needle or blood phobia.
17. Objection by the participant's GP to trial participation.

2.2. Dietary and Lifestyle Restrictions

Participants adhered to specific dietary and lifestyle restrictions throughout the study to minimise potential confounding factors and ensure safety. These restrictions included limitations on diet, substance use, and activities during inpatient stays and outpatient visits.

2.2.1. Dietary Restrictions

Standard meals and drinks were provided during inpatient stays, including meals approximately 4, 8, and 21 hours after dosing, as well as a snack 30 minutes post-dose. Specific dietary restrictions included:

- **Poppy Seeds:** Prohibited from 1 week before screening and each study session due to potential interference with tests.
- **Grapefruit Products:** Restricted from 7 days before dosing until the end of each inpatient stay (Part A only) to prevent interaction with drug metabolism.

2.2.2. Substance Restrictions

- **Alcohol:** Prohibited from 24 hours before admission until the end of each period of residence, and for 24 hours before each outpatient visit (including screening).
- **Caffeine:** Restricted during inpatient stays in Part A and from the morning of dosing until 4 hours post-dose in Part B.
- **Cannabis:** Prohibited for 24 hours before each study visit.
- **Smoking:** Not allowed from 72 hours before dosing until the morning after dosing in Part A and from 4 hours before until 4 hours after dosing in Part B.

2.2.3. Activity Restrictions

Participants were required to avoid strenuous exercise from 3 days before screening and from 3 days before admission until the end of each inpatient stay.

2.2.4. Contraceptive Requirements

Male participants were instructed not to father a child or donate sperm during the trial. There were no specific contraceptive requirements for male participants, consistent with Clinical Trials Facilitation and Coordination Group (CTFG) guidance and the SPL026 Investigator's Brochure (IB).

Female participants of childbearing potential were required to use an effective method of contraception from at least 28 days before their first dose until 24 hours after their last dose. Acceptable contraceptive methods included:

- Combined or progestogen-only hormonal contraception (oral, intravaginal, transdermal, or injectable) with ovulation inhibition.
- Progesterone-only oral hormonal contraception (even when ovulation inhibition was not the primary mode of action).
- Intrauterine device (IUD) or intrauterine hormone-releasing system (IUS).
- Bilateral tubal occlusion.
- Vasectomised partner (with medical confirmation of surgical success).
- Barrier methods, such as male or female condoms, with or without spermicide, or diaphragms/caps/sponges used with spermicide.

Women were classified as not being of childbearing potential if they met at least one of the following criteria:

- Post-menopausal, with the last menstrual period occurring at least 12 months ago and confirmed by follicle-stimulating hormone (FSH) testing at screening.
- Absence of uterus, ovaries, or fallopian tubes.

Women using hormone replacement therapy (HRT) could continue during the trial but were still required to use contraception.

Participants who practised true abstinence or were in same-sex relationships were exempt from contraceptive requirements, provided this aligned with their preferred and usual lifestyle. However, periodic abstinence (e.g., calendar, ovulation, symptothermal methods) and withdrawal were not considered acceptable contraceptive methods. If abstinence practices changed during the trial, participants were required to adopt one of the accepted methods of contraception.

2.3. Blinding

Due to the noticeable psychedelic effects of DMT fumarate, it was likely that both participants and investigators could determine whether a participant had received the active drug or placebo. Despite this, Stage 1 of the study adhered to double-blind principles, while Stage 2 was conducted as an open-label phase. MADRS assessments during both stages were carried out by an independent assessor who remained blinded and was not present during dosing or integration sessions.

The trial medication was repackaged and relabelled by the HMR Pharmacy according to the randomisation schedule. Active and placebo treatments were labelled in a way that made them indistinguishable. If the expiry dates of the placebo and active treatments differed, the labels used the earlier expiry date for both treatments to avoid identification. Each participant’s treatment was assigned a unique code number traceable to the medication batch number.

The active and placebo treatments were similar in appearance, with only slight differences in colour. To further maintain blinding, the HMR Pharmacy prepared syringes with the correct dosage volumes and obscured the contents with tape, ensuring that investigators could not discern any differences between the solutions. All participants in a group received the same volume of treatment to ensure consistency.

A sealed copy of the randomisation code was stored securely in the HMR Pharmacy, with another copy held by the bioanalytical laboratory. For emergencies, the investigator was provided with sealed envelopes containing treatment allocations for individual participants. These envelopes were stored in the trial master file and were readily accessible to clinical staff if needed.

While emergency procedures for unblinding were in place, no unblinding was required during the trial. Investigators, study psychiatrists, therapists, the Medical Monitor, and the Clinical Monitor remained blinded throughout the study.

2.4. Trial Compliance

The trial was conducted in compliance with The Medicines for Human Use (Clinical Trials) Regulations 2004[1] and The Human Medicines Regulations 2012[2], with current amendments; Good Manufacturing Practice (GMP)[3]; the SOPs issued by the Research Ethics Service for RECs in the UK[4]; and Good Clinical Practice (GCP)[5], which has its origins in the Declaration of Helsinki. The optional 6 month follow-up for exploratory objectives was done at Imperial College London (London, UK) under the oversight of Small Pharma (London, UK).

2.5. Trial Visits

2.5.1. Screening

Participants underwent comprehensive screening and preparation prior to dosing. Pre-screening occurred up to six months before dosing and involved remote assessments, including correspondence, phone, or video calls, to determine initial eligibility. Formal screening began up to three months before the first dose at Visit 1, including psychiatric assessment and a preparation session with the study psychiatrist and therapist. This session lasted up to one hour and was primarily a psychoeducational session relating to the psychedelic experience and the support provided in the trial. It was an opportunity to further build rapport and begin to explore expectations and intentions of the participant. There was sufficient opportunity for the participant to ask questions. Discontinuation of antidepressant medication was discussed where applicable. Weekly brief (10-15 mins) check-in calls were conducted by the study psychiatrist, while the participant was weaning off medication, to assess their mental state, risk and tolerance of medication discontinuation.

2.5.2. Main Trial

Visit 2 (Stage 1 dose). Participants were resident on the ward from the day before their dose (Day –1; baseline) until the morning after dosing (Day 2). A further preparation session and baseline assessments were made on Day –1 (including MADRS). The dose was administered on Day 1. Dosing was preceded by a therapist-guided final preparation session, and followed by completion of psychometric scales (see Table S1) and an integration session. Patients were discharged on Day 2 following a second integration session, physical examination and vital signs. They had follow-up assessments by telephone or video call on Day 8 (including MADRS assessment by an independent, blinded assessor).

Visit 3 (integration and follow-up for Stage 1 and admission to Stage 2). Participants attended the ward 13 days after their Stage 1 dose (Day 14). They underwent MADRS assessment (by an independent assessor) and a psychological assessment and discuss their Stage 1 experience with a psychiatrist. Based on these, and previous conversations with the participant, the psychiatrist will confirm if the patient may continue into Stage 2. If the patient proceeded to Stage 2, they received a dose of DMT on the morning of Day 15 and remained resident until the morning after their second dose (Day 16). As in Stage 1, dosing was followed by completion of psychometric scales (see Table S1) and an integration session. Patients were discharged on Day 16 following a second integration session, physical examination and vital signs. They had follow-up assessments by telephone or video call on Day 22 (including MADRS assessment by an independent, blinded assessor).

Visit 4 (integration and follow-up for Stage 2): patients returned to the ward for follow-up assessments 14 days after Visit 3 (Day 29 ± 2 days). This visit could be done video call.

Participants had final follow-up assessments via video call at 1 month (Day 45 ± 2 days) and 3 months (Day 105 ± 5 days) after Visit 3 (Day 15). After the final 3 month follow up visit for the final participant, the study was declared complete (December 2022) and the database was locked. In addition, some participants had an optional additional follow-up assessment at 6 months after Visit 3 (Day 15).

2.5.3. Optional Exploratory 6-month follow-up

The 6-month call was planned to be 3 months (91 days) after the final Day 105 follow-up call. This would be at study Day 196. The protocol and SOP did not specify an allowed deviation window for the 6-month call, because it was performed out-of-study and the endpoints were exploratory. On average, the calls took place at study Day 224. With a range of -5 to 134 days difference between the planned day and actual day. The average of Day 224 has been used here.

A delegated member of the ICL team arranged a video call with participants who had consented to being contacted in their original study ICF. The calls consisted of a general conversation about how the participant has been since their last follow-up (Day 105, 3 month); the administration of 2 questionnaires – the WEMWBS and the STAI-T; and also included additional questions during the interview covering their trial experience and how they have felt since, which will be used for qualitative analysis. The MADRS, using the SIGMA guide, was also administered separately from the interview, by a trained member of the ICL staff.

2.6. SPL026 (DMT fumarate) Drug Product

Small Pharma provided HMR with interim QP certification from the manufacturer, a certificate of analysis for the test product, and any other documents and data required by HMR's Qualified Person to release batches of IMP.

SPL026 (DMT fumarate) drug substance and drug product was manufactured in accordance with Good Manufacturing Practice (GMP) in the UK.

SPL026 drug product was supplied to the HMR Pharmacy in 10 mL clear glass vials containing 2.5 mg/mL (as free base) SPL026 in 10 mL of an aqueous sterile solution. Each vial had an identity label and was packaged in black Correx cartons (up to 80 per carton). The vials were dispensed as subject-specific doses by HMR Pharmacy, administered by syringe.

The drug was administered by continuous IV infusion via a single cannula using 2 syringes and 2 syringe pumps, joined by a 3-way tap. The first syringe pump infused 6 mg over 5 min (phase 1 of the infusion), followed by 15.5 mg infused from the second syringe and pump over 5 min (phase 2).

The placebo solution consisted of the same ingredients as the SPL026 drug product formulation, minus the active ingredient, although the ratio of those ingredients is slightly different to ensure the same pH and osmolality.

The HMR Pharmacy sent the repackaged and relabelled trial medication to MAC for patients to be dosed.

2.7. Therapeutic Support

The psychological support provided in this trial represented a time-limited, relational psychotherapeutic framework, based around the concept of psychological flexibility, and built on the foundation of a person's life history; A process that prioritises therapist presence and attunement to the individual's psychedelic experience [6].

Screening

Therapists were involved with the (psychiatrist-led) pre-screen video call for assessing suitability. During the in-person screening day, the therapists met with the participant to build trust and rapport, before providing the psychoeducational session.

Preparation

On the day prior to dosing, patients attended an interactive preparation session in the dosing room with the two therapists. This session lasted up to 90 minutes and involved orientating the participant to the setting and trial logistics. A visualisation exercise was led by the therapist, designed to provide experiential preparation for the psychedelic experience and support the negotiation of safe physical contact and grounding. Checking the patients' consent, readiness, and fostering their sense of agency, were crucial aspects of preparation. The potential for placebo was acknowledged, with emphasis on approaching every type of experience with equal therapeutic regard.

Dosing Day

On the dosing day, there was a further 45 minutes preparation session, immediately prior to dosing, to aid relaxation/readiness and support anticipatory anxiety where it arose. Therapists avoided overwhelming participants with more information. Participants were encouraged to be curious and invite self-compassion around their experiences. A guided body scan visualisation was read to the participant before a further check of their consent to go ahead with the infusion. Interpersonal grounding, using non-romantic hand/forearm holding, was offered and usually accepted. Participants were encouraged to fully immerse themselves in their internal experience, for as long as they needed, with no expectation to report back to the accompanying therapists.

Participants wore noise cancelling headphones and eyeshades. A specially curated, ambient, non-verbal music track was used during the dosing session. It was mixed by Max Cooper (music producer) and designed to follow the trajectory of the DMT experience. The tone of the music around the peak of the experience was expansive and this evolved into a more gentle and calming tone in the latter stages. There was consistency in the music across all participants. At all other times, ambient field recordings involving bird song were played in the room to contribute to a calming environment.

During the dosing, the therapists' primary role was to support the unfolding process of the participant's own psychedelic experience, without influencing it. Therapists provided co-regulation with curiosity, presence and humility. The study psychiatrist was either present in the room during dosing (when supporting as a co-therapist) or in an adjoining room for the full duration of the infusion session. They remained onsite until a further participant review around 4 hours after the infusion.

Room Setting

The dosing sessions were conducted in a dedicated clinical room adapted to support psychedelic experiences. The environment was designed to be calming and non-clinical, featuring soft lighting, natural imagery on the walls, salt lamps, and comfortable furnishings. Efforts were made to minimize medical cues and promote a sense of psychological safety and containment during the session. Some pictures of the dosing rooms can be found below. Photo credit Dr Graham Campbell, study psychiatrist.

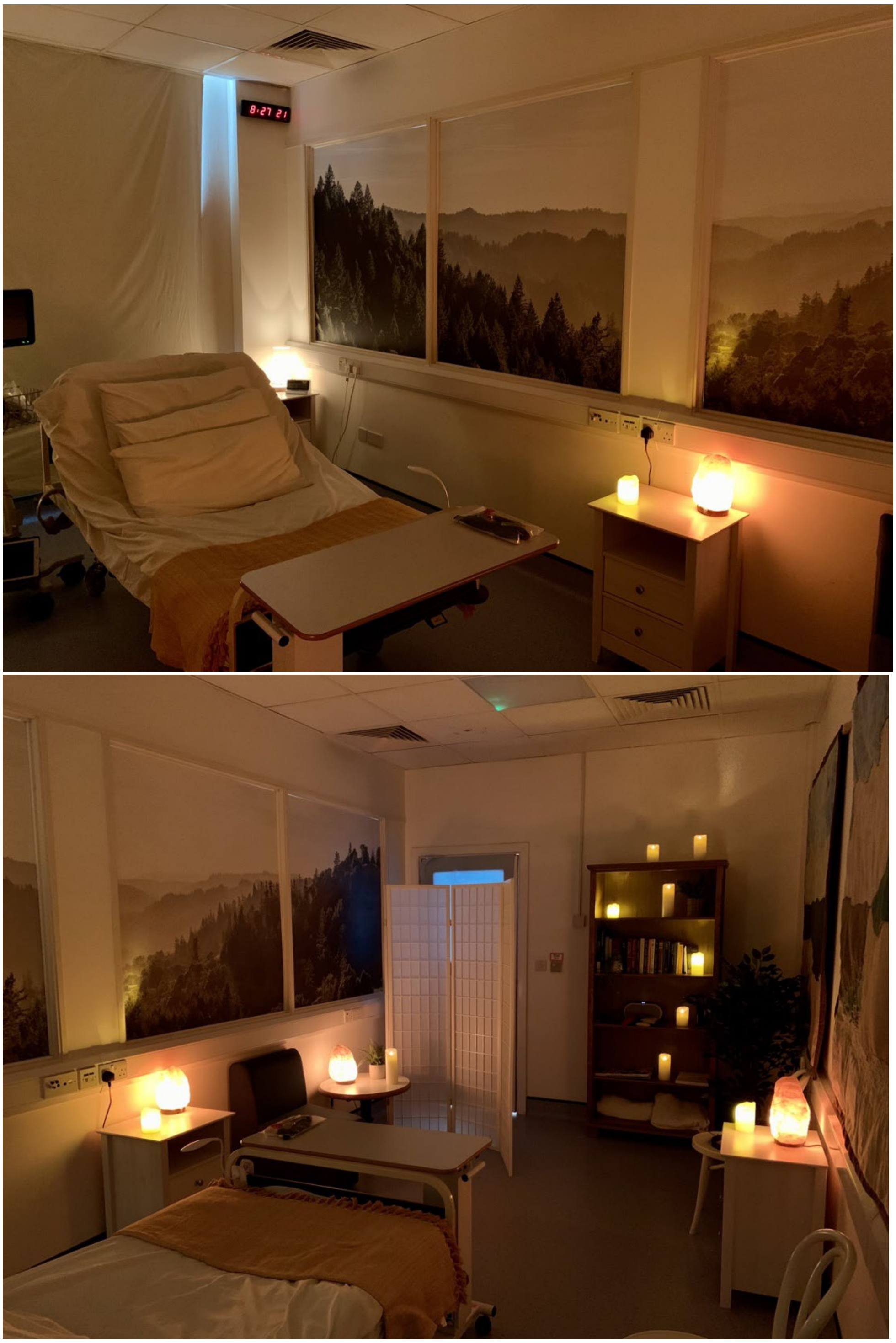

Figure S1: Dosing room

Integration

The therapeutic framework was developed from Watts & Luoma's 'ACE model' [6] and therapist experience of psilocybin trials at Imperial College London. It supports an emergent, participant-led integration process whilst bringing awareness to the transpersonal, relational, acceptance, connection and embodied elements of the psychedelic experience.

Rather than fixing meaning too quickly, the employed therapeutic approach encourages open curiosity and exploration of insights gleaned during the psychedelic experience. Therapists provide a non-directive, emotionally supportive presence, helping to manage distress and any adverse events. Participants were supported to use newfound insights in their daily life to support lasting, meaningful change.

Dosing day (Day 1) and Day 2 integration sessions were conducted in person at the trial site. Two therapists were present for integration and the sessions lasted up to an hour. In the Day 1 integration session, participants were encouraged to recall their experience in as much detail as possible. The Day 2 integration session was generally a more reflective discussion whilst revisiting and expanding on elements of the experience that held more meaning for the participant.

Follow-up

A third integration session was scheduled for Day 15. This was a further opportunity to discuss the participants' integration process in their everyday lives. Further check-in calls on Days 8, 29, 45, and 105 were conducted to support participants, identify and review any adverse events, and encourage completion of all questionnaires, including the Beck Suicide Scale. If a patient continued to discuss the content of their psychedelic experience during check-in calls, the therapists provided necessary integration support. On Day 105, at the end of the trial for each participant, information was given about psychedelic integration groups in the community and 6-month (Day 224) follow-up calls from the Imperial College team.

2.8. Complete list of collected measures

Assessment	Endpoint
Safety measures	
BSS	Secondary
Outcome measures (Efficacy)	
MADRS (2 weeks after the first dose)	Primary
MADRS (1 week after the first dose; all other timepoints after the second dose)	Secondary
Beck Depression Inventory II	Secondary
Spielberger's State-Trait Anxiety Inventory TRAIT subscale (STAI-T)	Secondary
Pharmacodynamic Outcome measures	
Warwick-Edinburgh Mental Wellbeing Scale (WEMWBS)	Secondary

Assessment	Endpoint
The Brief Experiential Avoidance Questionnaire (BEAQ)	Secondary
Profile of Mood States (POMS)	Secondary
Openness enriched 64-item Big Five Inventory (BFI)	Secondary
The Gratitude Questionnaire Six-Item Form (GQ-6)	Secondary
Snaith Hamilton Anhedonia Pleasure Scale (SHAPS)	Secondary
Flourishing Scale (FS-8)	Secondary
Life Orientation Test (LOT)	Secondary
Meaning in Life Questionnaire (MLQ)	Secondary
Brief Resilience Scale (BRS)	Secondary
Dysfunctional Attitude Scale (DAS)	Secondary
Ruminative Responses Scale (RRS)	Secondary
Barrett Impulsivity Scale (BIS)	Secondary
Social Connectedness Scale (SCS)	Secondary
Comprehensive assessment of Acceptance and Commitment (CompACT) Scale	Secondary
Work and Social Adjustment Scale (WSAS)	Secondary
Metaphysical Beliefs Scale (MBS)	Exploratory
Watts Connectedness Scale (WCS)	Exploratory
Psychological Insight Scale	Exploratory
Post-treatment Changes Scale (PTCS)	Exploratory
Predictor measures	
The Psychedelic Predictor Scale (PPS)	Exploratory
Subjective experience evaluation (acute effects)	
Mystical Experience Questionnaire (MEQ)	Exploratory
The Ego Dissolution Inventory (EDI)	Exploratory
Emotional Breakthrough Inventory (EBI)	Exploratory
Challenging Experience Questionnaire (CEQ)	Exploratory
5 Dimension Altered States of Consciousness Questionnaire (5D-ASCQ)	Exploratory
Exploratory visual analogue scales (VAS) (includes participant-led and physician-led Intensity Rating VAS [IRVAS])	Exploratory

Assessment	Endpoint
Metaphysical Experience Questionnaire (MPEQ)	Exploratory

Table S1: All Scales and questionnaires
Complete list of psychometric scale, depression rating scale, and questionnaire endpoints included in the trial, classified as primary, secondary or exploratory respectively

2.9. Additional Adverse Event Collection Methods

Investigators questioned participants about AEs using non-leading prompts, such as "How are you feeling?", and also recorded spontaneously reported events and clinically significant findings from physical examinations or objective tests (e.g., laboratory variables, ECG). Events linked to the psychedelic effects of DMT, such as visual perceptual changes, auditory or tactile hallucinations, ego dissolution, and time-perception changes, were not classified as AEs if they occurred during the dosing period, fully resolved within 45–60 minutes, and were tolerated by the participant. These events, aligned with the intended mechanism of action of DMT fumarate, were documented using psychometric scales completed immediately post-dose and therapists' notes.

2.10. Sample Size - Rationale

The sample size calculation was based on a two-sided, two-sample t-test with equal variance at a significance level of 0.05 and a 1:1 allocation ratio, using data from Palhano-Fontes et al. (2019), an existing study investigating the antidepressant effects of the DMT containing brew Ayahuasca. A sample size of 28 to 36 participants provided 80% to 90% power to detect a 12.5-point mean difference in MADRS score change from baseline.

Table S2 displays sample size requirements to detect a difference in mean change from baseline at Day 7 in QIDS, HAM-D and MADRS between active and placebo based on a 2-sided two-sample equal-variance t-test conducted at the 5% level of significance and a 1:1 allocation ratio. Estimates for the standard deviation for HAM-D and MADRS are taken from Palhano-Fontes, et al., 2018, where the mean difference in the change from baseline at Day 7 between active and placebo was 9.7 for HAM-D and 13.9 for MADRS. The estimate for standard deviation for QIDS was supplied by Dr Erritizoe, and was estimated from the change form baseline to Week 5.

Endpoint	Mean Difference	Standard Deviation	Power	Sample Size
QIDS	5	5.6	80%	42
QIDS	5	5.6	90%	56
HAM-D	5	8.2	80%	88
HAM-D	5	8.2	90%	116
HAM-D	7.5	8.2	80%	40
HAM-D	7.5	8.2	90%	54
HAM-D	10	8.2	80%	24
HAM-D	10	8.2	90%	32
MADRS	10	11.0	80%	42
MADRS	10	11.0	90%	54
MADRS	12.5	11.0	80%	28
MADRS	12.5	11.0	90%	36
MADRS	15	11.0	80%	20
MADRS	15	11.0	90%	26

Table S2: Sample size requirements

2.11. Handling of Missing Data

All participants who withdrew prematurely from the study were included in the statistical analyses to maintain a comprehensive dataset. For participants who completed treatment but had missing data, the nature and extent of the missing data were recorded in the individual participant listings.

Missing data were imputed only for secondary and exploratory psychometric scales and questionnaires, including efficacy measures, using a person-mean imputation method. This approach replaced missing responses with the mean of the participant's available responses, provided at least 90% of the items for a given scale or subscale were completed. Missing data for primary endpoints and safety measures were not imputed and were treated as missing.

Data collected at unscheduled time points were excluded from analyses but included in individual participant listings for transparency. For adverse events (AEs) or concomitant medication reports, if time details (hours or minutes) were missing but the day was recorded, the time was calculated in days. When date information was incomplete or missing, derived times (e.g., AE onset relative to the last dose) were marked as missing.

3. Supplementary Results

3.1. Consort Diagram

From HMR (data not available from MAC), approximately 6900 MDD patients expressed interest in study participation. Of these, 3363 were sent information but did not respond; 2552 were not suitable; 119 were not interested; 470 were on medication with not sufficient time to be weaned off; 89 were rejected based on GP reply; and 299 entered prescreening. Across both sites, 399 signed a prescreening consent form and were prescreened.

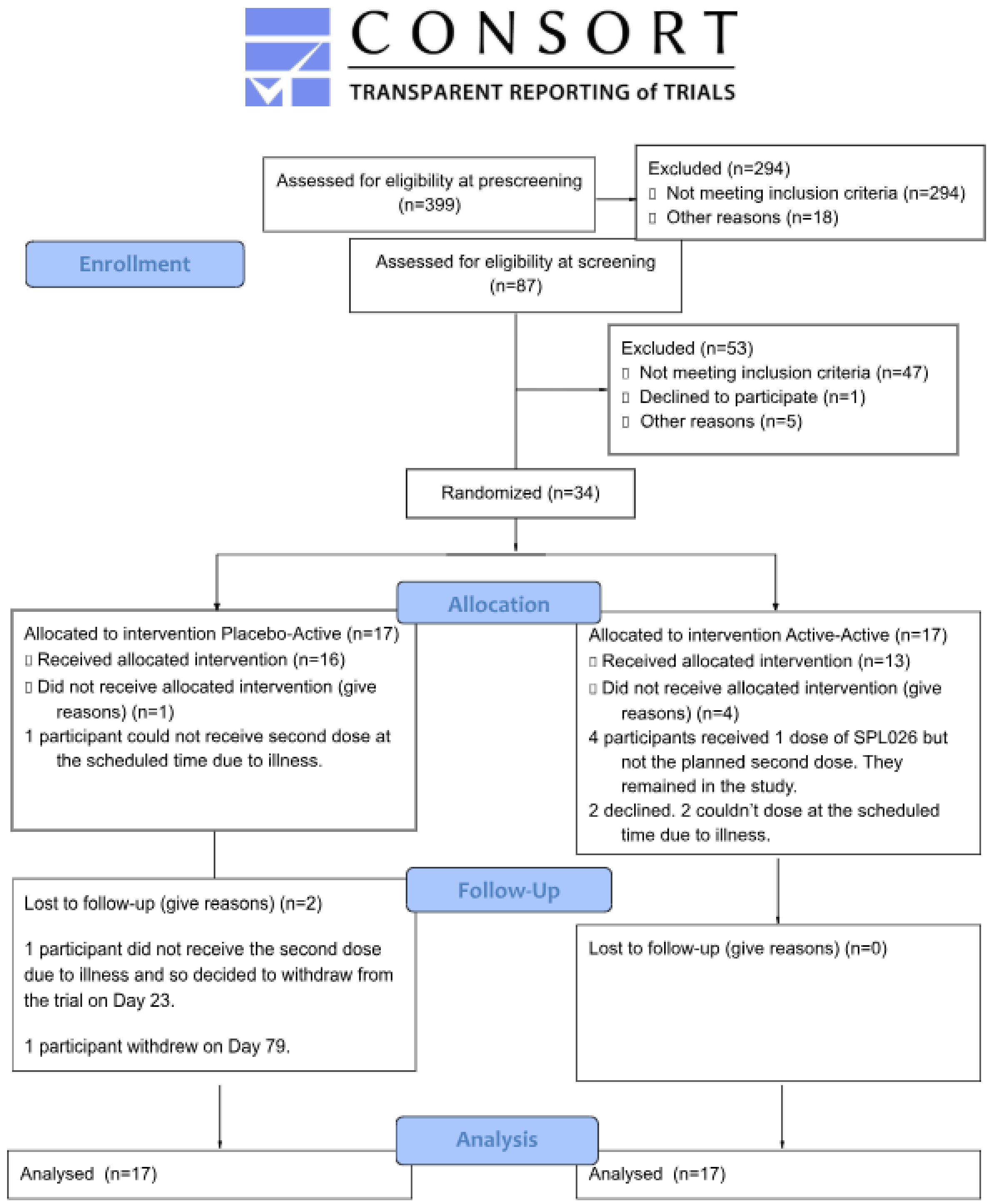

Figure S2: CONSORT flow diagram

3.2. Previous and concomitant medication

3.2.1. Overview

Participants' previous and concomitant medication usage was carefully monitored throughout the trial to ensure protocol compliance and data integrity. None of the

concomitant medications administered during the trial were deemed by investigators to have influenced the study results.

3.2.2. Previous Medications

At the time of screening, eight participants were taking psychiatric medications, which were discontinued before their first dosing session. These included antidepressants such as sertraline and citalopram. Participants required to discontinue antidepressants underwent a washout period to ensure complete clearance before receiving the study treatment.

To manage potential withdrawal symptoms, participants discontinuing psychiatric medications were closely monitored by the study psychiatrist through outpatient visits, phone calls, or video calls. The shortened BSS was administered weekly during this period. After completing the final dosing session and the 2-week post-dose assessment, participants could resume antidepressant medication, if needed, as coordinated by the study psychiatrist and their GP.

3.2.3. Concomitant Medications During the Trial

A total of 23 participants used concomitant medications during the trial. These were recorded in the eCRF, along with dosage, duration, and reasons for administration. Key examples include:

Antidepressant Medications:

- Four participants resumed antidepressant treatments during the trial:
    - Escitalopram (10 mg QD from Day 68 onward).
    - Sertraline (50 mg QD for 21 days starting Day 39, increased to 100 mg QD from Day 60 onward).
    - Citalopram (5 mg QD from Day 34 onward).
    - Venlafaxine (37.5 mg BID for four days starting Day 96).

Anxiolytic Medications:

- Three participants received medication for anxiety, including propranolol, diazepam, and promethazine.

Analgesics for TEAEs:

- Thirteen participants used oral analgesics for treatment-emergent adverse events, including:
    - Paracetamol (up to 1 g): Administered for headaches, muscle tension, or viral illness.
    - Ibuprofen (up to 500 mg): Taken for muscle tension, chest pain, or sore throat.
    - Other analgesics included aspirin, co-codamol (paracetamol with codeine), naproxen, and morphine.

Other Concomitant Medications:

- Antihistamines: Two participants took loratadine or cetirizine for hay fever symptoms.
- Vitamins and Supplements: Two participants used vitamin C, vitamin D, magnesium, or multivitamins for general health or cold symptoms.

3.2.4. Adverse Event-Driven Medications

Medications were also administered to address specific adverse events during the study. For example, one participant who experienced a fractured radius and ulna received treatment that included intravenous ketamine, morphine (IV and oral), and codeine, in addition to paracetamol and ibuprofen.

Further details of medications administered, including exact dosages and timing, are available in the corresponding clinical listings.

3.3. Participant Withdrawals

Participants could withdraw from the trial at any time without providing a reason. Investigators could also withdraw participants for medical reasons, including intolerance to trial medication, intercurrent illness, the need for contraindicated medication, or withdrawal of consent.

3.3.1. Withdrawals during the trial

1. One participant withdrew after experiencing a viral infection deemed unrelated to treatment.
2. Another participant withdrew due to personal concerns about recalling their dosing experience.

No participants who withdrew were replaced in the trial. Investigators assessed the reasons for withdrawal, recorded the circumstances and medical details in the electronic case report form (eCRF), and invited withdrawn participants to consent to follow-up examinations.

3.3.2. Subjects not receiving a second dose

4 participants in AA group did not receive their second doses but remained in the trial (thus referred to as A-). For one participant this was due to therapists being ill and replacement not being possible. One participant reported that the first DMT experience was so intense they did not wish for an additional session 2 weeks later, and one expressed some fear/anxiety about having their second dose and therefore opted out. The last participant who opted out of their second DMT said she did not need it after a powerful first experience with MADRS score drop from 34 pre-dose to 5 at Day 14.

3.3.3. Follow-Up for Withdrawn Participants

Withdrawn participants who consented underwent standard medical examinations and laboratory tests that would have been conducted at the 2-week post-dose outpatient visit (Day 14 or 29, depending on the number of doses received). These results were recorded in the eCRF. Participants in later study stages were also asked to consent to follow-up assessments at 1 and 3 months post-dose to ensure their safety and well-being.

3.3.4. Immediate Discontinuation of Treatment

A participant’s infusion was stopped immediately if:

- The participant expressed a desire to discontinue.
- The study psychiatrist deemed it unsafe to continue.

Procedures for withdrawal from treatment, including safety measures, were outlined in the protocol. These were followed meticulously to ensure the safety and rights of all participants.

3.4. Protocol Deviations

Protocol deviations were categorised as major or minor based on their potential impact on the study outcomes.

3.4.1. Major Protocol Deviations

Three major deviations occurred, which led to the exclusion of certain data from the analysis:

1. **Delayed ECG Completion:** One participant had their 180-minute postdose ECG (Day 1) conducted one day late due to an error. The affected ECG data were excluded from analysis.
2. **Late Follow-Up Assessments:** One participant completed their Day 105 follow-up assessments (MADRS and BSS) 51 days outside the protocol-defined window, after missing two scheduled visits. These assessments were excluded from the analysis.
3. **Late Psychometric Questionnaire Completion:** One participant lost their Day 8 PD questionnaires, which were re-sent by post. Upon receipt, the participant completed the measures outside the deviation window without recording the specific completion date. These questionnaires were excluded from analysis due to timing discrepancies.

3.4.2. Minor Protocol Deviations

All other deviations were classified as minor and were determined not to impact data integrity. These included:

- **Dosing Deviations:** Five participants experienced delays or errors during infusion:
  - Four participants had infusions that ended later than scheduled due to late starts or technical issues with infusion lines.
  - One participant's infusion times were recorded inaccurately for both dosing sessions.
- **Lifestyle Restrictions:** Two participants violated study restrictions by consuming vitamin supplements or alcohol within restricted windows before follow-up visits.
- Assessment Timing and Completion Errors:
  - Some participants completed efficacy and safety assessments (e.g., MADRS, STAI-T, BDI-II, and BSS) outside the allowable windows or failed to complete them altogether.
  - In a few cases, follow-ups were conducted remotely due to participant or staff unavailability.
- Procedural Deviations:
  - Several vital signs, ECGs, PK samples, and other safety measurements were conducted outside the protocol-defined windows due to logistical challenges, such as courier delays or participant absence.
  - In total, 143 procedures across 31 participants were conducted outside the allowable time windows, with most deviations within 30 minutes. Only a few exceeded this threshold, such as local tolerability assessments (up to 3.2 hours late) or PK samples (up to 39 minutes late).
- **Informed Consent Errors:** Two participants signed an outdated version of the informed consent form during Day 1 admission. The error was rectified and did not impact the study.
- **Laboratory Testing Error:** Certain HIV test kits used during screening were later found to be faulty. Affected participants were contacted, and repeat tests were recommended where necessary.

These minor deviations were reviewed and deemed unlikely to affect study outcomes.

3.5. Secondary Analysis – all BDI and STAI-T data

Group	Stage	Treatment	Time	n	BDI-II Mean (SD)	BDI-II Change from baseline (SD)	n	STAI-T Mean (SD)	STAI-T Change from baseline (SD)
Placebo-Active (PA) group<br><br>(N=17)	Stage 1<br><br>(blinded)	Placebo<br><br>(N=17)	Day –1	16	32.8 (9.21)	–	17	63.1 (4.99)	–
			Day 8	–	ND	ND	16	61.1 (8.55)	–1.9 (4.66)
			Day 14	15	27.1 (11.91)	-3.7 (5.12)	16	59.3 (7.76)	–3.4 (4.73)
	Stage 2<br><br>(open-label)	DMT<br><br>(after placebo)<br><br>(N=16)	Day 22	–	ND	ND	15	50.6 (16.23)	–11.9 (14.22)
			Day 29	15	20.5 (16.18)	-11.4 (15.7)	15	52.5 (16.55)	–10.0 (14.11)
			Day 45	14	15.7 (12.58)	-15.2 (12.03)	14	50.6 (15.14)	–12.3 (12.65)
			Day 105	13	12.3 (13.7)	-19.4 (13.49)	13	49.2 (16.24)	–14.2 (14.21)
			Day 224	–	ND	ND	11	48.0 (13.78)	-14.5 (11.60)
Active-Active (AA) group<br><br>(N=17)	Stage 1<br><br>(blinded)	DMT<br><br>(N=17)	Day –1	17	33.6 (6.11)	–	17	65.2 (5.88)	–
			Day 8	–	ND	ND	13	51.7 (17.54)	–13.5 (16.39)
			Day 14	17	16.8 (13.21)	-16.8 (12.18)	17	54.2 (13.69)	–11.0 (12.87)
	Stage 2<br><br>(open-label)	DMT<br><br>(after active)<br><br>(N=13*)	Day 22	–	ND	ND	12	54.7 (14.88)	–10.3 (14.91)
			Day 29	11	13.1 (13.23)	-21.0 (12.62)	11	48.6 (13.89)	–16.2 (13.23)
			Day 45	10	14.8 (15.11)	-20.9 (12.75)	10	49.7 (15.30)	–16.5 (15.15)
			Day 105	10	19 (11.36)	-16.7 (8.68)	10	57.1 (14.70)	–9.1 (13.63)
			Day 224	–	ND	ND	9	51.3 (11.19)	-14.2 (9.32)
All participants (N=34)	Stage 2<br><br>(open-label)	DMT (1 or 2 doses (N=33)	Day 105	25	15.5 (13.57)	-18.8 (12.52)	26	51.8 (16.08)	-13.2 (15.04)
			Day 224	–	ND	ND	24	48.3 (14.17)	-16.25 (13.55)

Table S3: BDI-II and STAI-T Outcome data at all time points and in both stages
*) these AA data are without the 4 individuals (A-) who did not receive their second DMT dose. ND: Not done.

3.6. Secondary Analysis - MADRS Score Comparison Between PA and AA Groups in Stage 2

To evaluate the potential differences in clinical outcomes between one and two doses of DMT fumarate, MADRS scores were assessed at multiple time points: 1 week (Day 22), 2 weeks (Day 29), 4 weeks (Day 45), and 12 weeks (Day 105) following the second dose. The analysis aimed to determine whether a single dose administered during Stage 2 after placebo (PA group) could produce comparable long-term reductions in depression severity to the two-dose regimen received by the AA group across Stages 1 and 2. Results indicated

no statistically significant differences in MADRS scores between the groups at any of the assessed time points. While mean scores showed slight numerical variations, such as a greater reduction at earlier time points in the AA group compared to the PA group, these differences did not reach statistical significance. For example, at 1 week (Day 22), the mean difference in MADRS scores between the groups was -3.26 points (95% CI: -11.34 to 4.81; p = 0.42). Similarly, at later time points, including 12 weeks (Day 105), the mean difference was 7.03 points (95% CI: -1.04 to 15.11; p = 0.08), reflecting no clear advantage of the two-dose regimen.

Treatment Group	Time Point (Days)	LS means	SE	Mean Difference	P val	95% CI
Stage 2 after placebo; PA group (N=16)	Day 22	–10.48	2.75	–		–
	Day 29	–10.40	2.58	–		–
	Day 45	–12.90	2.58	–		–
	Day 105	–15.19	2.75	–		–
Stage 2 after active; AA group (N=13)	Day 22	–13.74	2.97	–3.26	0.42	(–11.34, 4.81)
	Day 29	–13.66	2.97	–3.26	0.41	(–11.10, 4.59)
	Day 45	–12.74	2.97	0.16	0.96	(–7.68, 8.00)
	Day 105	–8.16	2.97	7.03	0.08	(–1.04, 15.11)

Table S4: Comparison of MADRS score changes
Comparison of MADRS score changes at 1 week, 2 weeks, 1 month, and 3 months after the second dose between participants receiving a single DMT dose in Stage 2 after placebo (PA group) and those receiving two doses across Stages 1 and 2 (AA group). Results include LS means, SE, and 95% CI. Results assess whether a single dose in Stage 2 yields comparable long-term outcomes to the two-dose regimen.

3.7. Supplementary Analysis - AA Group Within-Participant Comparison of Two DMT Doses

To evaluate the potential additional benefit of a second DMT dose, changes in MADRS scores at 1 and 2 weeks after the first dose (Stage 1: Days 8 and 14) were compared with those following the second dose (Stage 2: Days 22 and 29) in participants receiving two DMT doses (AA group). At 2 weeks, reductions from baseline were -10.42 (SE: 2.35) following the first dose and -13.97 (SE: 2.80) after the second dose, with a mean difference of 3.55 points (95% CI: -3.87 to 10.98; p = 0.33), suggesting no additional effect of the second dose. The baseline used was Day -1 in all cases. Full results are shown in Table S5:
.

Treatment Group	Time Point (Days)	LS means	SE	1st DMT dose - 2nd DMT dose		
				Mean Difference	P val	95% CI
1st DMT dose in AA group (N=17)	Day 8	–12.35	2.42	–		–
	Day 14	–10.42	2.35	–		–
2nd DMT dose in AA group (N=13)	Day 22	–14.06	2.80	1.71	0.44	(–5.80, 9.21)
	Day 29	–13.97	2.80	3.55	0.33	(–3.87, 10.98)

Table S5: Within-participant comparison of MADRS score changes

Within-participant comparison of MADRS score changes at 1 and 2 weeks after the first dose (Days 8 and 14) and the second dose (Days 22 and 29) in the AA group to assess the possible additional benefit of the second dose. Results include LS means, SE, and 95% CI.

3.8. Supplementary Analysis - Timing of the First DMT Dose: Stage 1 vs Stage 2

This analysis assessed whether the timing of the first DMT dose influenced clinical outcomes by comparing MADRS score changes at 1 and 2 weeks between participants receiving their first dose during Stage 1 (AA group) and Stage 2 (PA group). At 2 weeks, reductions were -10.58 in Stage 1 and -11.03 in Stage 2, with a mean difference of 0.45 points (95% CI: -7.11 to 8.01; p = 0.69), indicating no significant influence of timing. The baseline used was Day -1 in all cases.

Treatment Group	Time Point (Days)	LS means	SE	Stage 1 - Stage 2		
				Mean Difference	P val	95% CI
Stage 1 after DMT, AA group (N=17)	Day 8	–12.50	2.64	–		–
	Day 14	–10.58	2.57	–		–
Stage 2 after DMT, PA group (N=16)	Day 22	–11.04	2.83	–1.45	0.71	(–9.39, 6.49)
	Day 29	–11.03	2.64	0.45	0.69	(–7.11, 8.01)

Table S6: Comparison of MADRS score changes at 1 and 2 weeks between participants
Comparison of MADRS score changes at 1 and 2 weeks between participants receiving their first DMT dose during the blinded phase (Stage 1, Days 8 and 14) and those receiving their first dose during the open-label phase (Stage 2, Days 22 and 29). Results include LS means, SE, mean differences, p-values, and 95% CI.

3.9. Supplementary Analysis - MADRS Remission Rates

This section provides supplementary information on MADRS remission rates over time, complementing the primary results presented in the main text. Remission was defined as a MADRS score ≤10. Table S7: presents remission rates for both PA and AA groups, along with the differences, standard errors (SE), and 95% confidence intervals (CI) for each time point. Total remission rates for all participants at Days 105 and 224 are also shown.

Time (Day)	PA Remitters, n (N)	AA Remitters, n (N)	% PA Remitters	% AA Remitters	% Difference	SE	Lower CI	Upper CI
Day 8	2 (16)	7 (16)	13%	44%	31.25	14.91	2.04	60.46
Day 14	2 (17)	5 (17)	12%	29%	17.65	13.53	-8.88	44.18
Day 22	6 (14)	7 (12)	43%	58%	15.48	19.43	-22.60	53.56
Day 29	5 (16)	6 (12)	31%	50%	18.75	18.51	-17.53	55.03
Day 45	8 (16)	6 (12)	50%	50%	0.00	19.09	-37.42	37.42
Day 105	8 (14)	4 (12)	57%	33%	-23.81	18.98	-61.00	13.38
Day 224	6 (12)	2 (9)	50%	22%	-27.78	20.01	-67.00	11.44
Day 105 (all)	14 (30)		47%					

Day 224 (all)	10 (25)	40%

Table S7: MADRS Remission Rates Over Time

3.10. Supplementary Materials – Acute Measures and moderation effects of Mystical Experience (MEQ) Scores and related measures of the acute psychedelic experience

3.10.1. Acute measures

3.10.1.1. Mystical Experience Questionnaire (MEQ-30)

Scale	Treatment	N	Mean	SD	Median	Min	Max
Mystical	Placebo	17	0.149	0.2892	0	0	1.2
Mystical	21.5 mg DMT (Stage 1)	17	2.702	1.0312	2.733	1.2	4.53
Mystical	21.5 mg DMT (Stage 2 after Placebo)	16	1.817	1.4046	1.4	0	4.27
Mystical	21.5 mg DMT (Stage 2 after Active)	13	3.503	1.0423	3.8	1.4	5
Positive Mood	Placebo	17	0.784	0.7834	0.5	0	2.83
Positive Mood	21.5 mg DMT (Stage 1)	17	3.471	0.9705	3.667	1.5	5
Positive Mood	21.5 mg DMT (Stage 2 after Placebo)	16	2.531	1.3392	2.5	0.33	4.67
Positive Mood	21.5 mg DMT (Stage 2 after Active)	13	3.628	1.11	3.833	1.5	5
Transcendence	Placebo	17	0.412	0.5274	0	0	1.33
Transcendence	21.5 mg DMT (Stage 1)	17	4.039	0.7895	4.167	2.17	5
Transcendence	21.5 mg DMT (Stage 2 after Placebo)	16	3.5	0.9389	3.417	1.83	4.67
Transcendence	21.5 mg DMT (Stage 2 after Active)	13	4.333	0.6273	4.5	3	5
Ineffability	Placebo	17	0.196	0.3547	0	0	1
Ineffability	21.5 mg DMT (Stage 1)	17	4.314	0.8289	4.667	2.33	5
Ineffability	21.5 mg DMT (Stage 2 after Placebo)	16	3.688	1.4426	4.167	0.33	5
Ineffability	21.5 mg DMT (Stage 2 after Active)	13	4.513	0.6752	5	3.33	5
**Total MEQ Score**	Placebo	17	0.333	0.3385	0.2	0	1.1
**Total MEQ Score**	21.5 mg DMT (Stage 1)	17	3.284	0.7381	3.267	2.03	4.6
**Total MEQ Score**	21.5 mg DMT (Stage 2 after Placebo)	16	2.483	1.1588	2.4	0.83	4.4
**Total MEQ Score**	21.5 mg DMT (Stage 2 after Active)	13	3.795	0.8519	4	1.93	5

NB: The maximum mean score is 5.

Table S8: MEQ-30 scale scores
(Mystical, Positive Mood, Transcendence, Ineffability, and Total) by treatment arm: placebo, DMT Stage 1, and DMT Stage 2 following placebo or active

Figure S3: Mystical Experience Questionnaire (MEQ-30) total scores across placebo and DMT conditions (Stage 1 and Stage 2)

3.10.1.2. 11-D Altered States of Consciousness (11D-ASC)

Factor	Treatment	N	Mean	SD	Median	Min	Max
Experience of Unity	Placebo	17	3.25	6.31	0.00	0.00	21.60
Experience of Unity	21.5 mg DMT (Stage 2 after Placebo)	16	38.01	33.06	34.70	0.00	84.20
Experience of Unity	21.5 mg DMT (Stage 1)	17	51.02	22.08	50.40	3.00	89.00
Experience of Unity	21.5 mg DMT (Stage 2 after Active)	13	60.19	25.01	65.00	23.60	100.00
Spiritual Experience	Placebo	17	2.71	4.81	0.00	0.00	17.00
Spiritual Experience	21.5 mg DMT (Stage 2 after Placebo)	16	36.04	22.28	40.67	0.00	75.00
Spiritual Experience	21.5 mg DMT (Stage 1)	17	58.22	22.80	56.33	25.00	96.00
Spiritual Experience	21.5 mg DMT (Stage 2 after Active)	13	61.44	28.10	68.00	1.00	100.00
Blissful State	Placebo	17	6.75	14.27	1.00	0.00	57.67
Blissful State	21.5 mg DMT (Stage 2 after Placebo)	16	33.21	30.91	30.50	0.00	89.00
Blissful State	21.5 mg DMT (Stage 1)	17	44.78	26.11	44.00	5.67	90.33
Blissful State	21.5 mg DMT (Stage 2 after Active)	13	54.82	33.99	62.67	0.00	100.00
Insightfulness	Placebo	17	5.51	9.13	0.67	0.00	26.33
Insightfulness	21.5 mg DMT (Stage 2 after Placebo)	16	36.46	27.20	38.33	0.00	82.00
Insightfulness	21.5 mg DMT (Stage 1)	17	50.31	22.21	44.33	12.00	87.00
Insightfulness	21.5 mg DMT (Stage 2 after Active)	13	55.46	23.51	55.67	13.33	100.00
Disembodiment	Placebo	17	1.47	3.44	0.00	0.00	13.33
Disembodiment	21.5 mg DMT (Stage 2 after Placebo)	16	48.56	36.44	48.83	0.00	100.00
Disembodiment	21.5 mg DMT (Stage 1)	17	70.65	23.73	70.00	5.00	100.00
Disembodiment	21.5 mg DMT (Stage 2 after Active)	13	56.03	27.22	52.33	0.67	100.00

Impaired Control and Cognition	Placebo	17	1.74	2.80	0.14	0.00	8.86
Impaired Control and Cognition	21.5 mg DMT (Stage 2 after Placebo)	16	36.59	23.95	38.43	0.00	79.29
Impaired Control and Cognition	21.5 mg DMT (Stage 1)	17	40.47	19.07	37.57	8.57	69.29
Impaired Control and Cognition	21.5 mg DMT (Stage 2 after Active)	13	39.13	16.01	42.86	15.57	59.71
Anxiety	Placebo	17	1.19	2.16	0.17	0.00	7.17
Anxiety	21.5 mg DMT (Stage 2 after Placebo)	16	44.97	29.72	42.00	0.00	96.83
Anxiety	21.5 mg DMT (Stage 1)	17	47.11	23.53	37.33	9.83	82.00
Anxiety	21.5 mg DMT (Stage 2 after Active)	13	44.30	24.61	45.00	4.83	78.33
Complex Imagery	Placebo	17	7.41	15.15	0.67	0.00	53.33
Complex Imagery	21.5 mg DMT (Stage 2 after Placebo)	16	60.94	32.00	68.50	0.00	100.00
Complex Imagery	21.5 mg DMT (Stage 1)	17	73.35	16.90	70.33	40.67	98.00
Complex Imagery	21.5 mg DMT (Stage 2 after Active)	13	69.72	12.66	66.67	54.33	100.00
Elementary Imagery	Placebo	17	14.00	20.12	2.33	0.00	65.67
Elementary Imagery	21.5 mg DMT (Stage 2 after Placebo)	16	71.77	27.50	77.33	17.00	100.00
Elementary Imagery	21.5 mg DMT (Stage 1)	17	89.75	13.33	93.33	53.33	100.00
Elementary Imagery	21.5 mg DMT (Stage 2 after Active)	13	89.46	11.34	94.00	70.33	100.00
Audio-Visual Synesthesiae	Placebo	17	9.35	18.51	0.33	0.00	70.33
Audio-Visual Synesthesiae	21.5 mg DMT (Stage 2 after Placebo)	16	64.77	32.47	73.50	0.00	100.00
Audio-Visual Synesthesiae	21.5 mg DMT (Stage 1)	17	63.61	35.16	80.67	3.33	100.00
Audio-Visual Synesthesiae	21.5 mg DMT (Stage 2 after Active)	13	60.31	29.11	68.00	0.00	91.67
Changed Meaning of Percepts	Placebo	17	4.29	10.97	0.67	0.00	46.00
Changed Meaning of Percepts	21.5 mg DMT (Stage 2 after Placebo)	16	36.73	30.18	37.17	0.00	90.33
Changed Meaning of Percepts	21.5 mg DMT (Stage 1)	17	41.26	24.91	38.67	0.00	78.33
Changed Meaning of Percepts	21.5 mg DMT (Stage 2 after Active)	13	36.80	23.50	34.00	4.00	86.00
**Average 5D-ASC Score**	Placebo	17	5.06	5.77	2.04	0.03	20.49
**Average 5D-ASC Score**	21.5 mg DMT (Stage 2 after Placebo)	16	38.22	17.78	40.18	5.34	64.30
**Average 5D-ASC Score**	21.5 mg DMT (Stage 1)	17	43.72	8.58	44.18	22.19	58.99
**Average 5D-ASC Score**	21.5 mg DMT (Stage 2 after Active)	13	44.11	10.11	46.05	28.35	61.23

Table S9: Altered States of Consciousness Questionnaire (11D-ASC) factor and total scores across placebo and DMT treatment conditions

Figure S4: Altered States of Consciousness Questionnaire (11D-ASC) factor scores for placebo and DMT treatment (Stage 1 and Stage 2)

3.10.2. Mystical Experience Questionnaire (MEQ) - Moderation Analyses

The full sample was used to test the moderating influence of *Mystical Experience Questionnaire)* on antidepressive response two weeks following participants' first active DMT dose. As justified by the observation of no MADRS change difference between individuals who had their first active dose following a placebo (PA group) versus without an initial placebo (AA group), data was consolidated such that Day -1 and Day 14 *MADRS* scores for AA participants were used; Day 14 and Day 28 *MADRS* scores for PA participants were used; and Day 1 and Day 15 *MEQ* scores were used for AA and PA participants, respectively. Linear mixed effects models were conducted in which *Depression* scores were regressed onto the interaction between *Time* (levels: 1 day pre-dose, 2 weeks post-dose) and *Mystical Experience*. A random intercept effect was included in the models which controls for baseline *Depression*. Unstandardized interaction term coefficients (B) represent the change in depressive response associated with being one standard-deviation higher in *Mystical Experience*.

Antidepressant change two weeks following participants' first active DMT dose was observed to be moderated by their *Mystical Experience* scores. Specifically, being one-standard-deviation higher in *Mystical Experience* was associated with an incremental 5.56 unit decrease in *MADRS* scores two weeks later. Figure S4 illustrates that participants with higher baseline depression were no more likely to report mystical-type experience than patients lower in depression, but patients who reported stronger levels were significantly more likely to exhibit decreases in depressive symptoms two weeks following their first DMT dose.

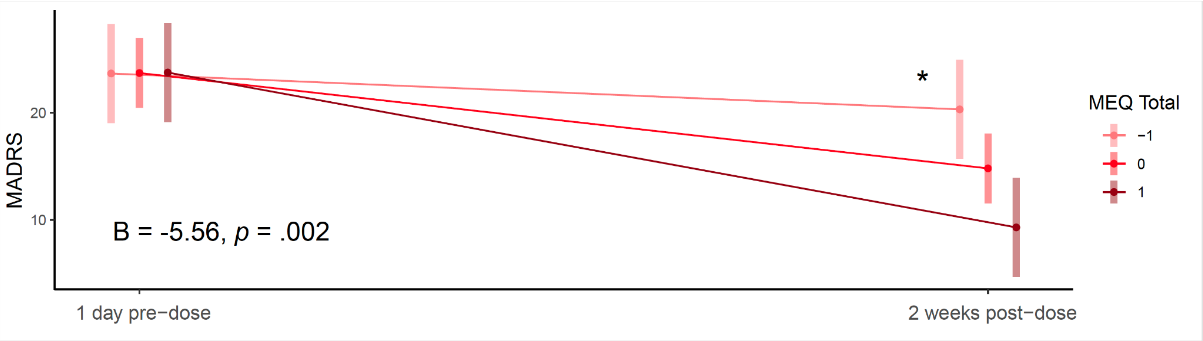

Figure S5: Line plot illustrating the moderating effect of Mystical Experience
Specifically, the plot shows trajectories of antidepressive response two weeks post-dose at different levels of MEQ Total in the full sample. MEQ Total scores from participants' first active DMT dose were used, and MADRS scores from one day before the first DMT dose and two weeks following the first DMT dose were used. -1, 0, and 1 refer to one standard deviation below mean MEQ Total (-1), mean MEQ Total (0), and one standard deviation above mean MEQ Total. *$p < .005$.

3.10.3. Other measures of the acute subjective psychedelic experience - Moderation and Correlation Analyses

To complement the MEQ moderation analysis, exploratory models were run to assess whether other acute experience measures moderated antidepressant effects at 2 weeks following the first active DMT dose.

Among subscales of the 11D-ASC (7), only the Unity dimension significantly moderated antidepressant response (B = –5.27, p = 0.006), with higher scores associated with greater reductions in MADRS. Specifically, being one standard deviation higher in Unity was associated with an incremental 5.27-point decrease in depression severity two weeks post-dose. The Ego Dissolution Inventory (EDI; 8) showed a significant interaction (B = –5.78, p = 0.002), where each standard deviation increase in ego dissolution corresponded to a 5.78-point additional reduction in MADRS scores. “Ego dissolution” and “Unity” constructs are both closely related to mystical experiences, further supporting the MEQ findings outlined in the previous paragraph. Other subscales, including Spiritual Experience, Blissful State, and Disembodiment, did not show significant moderation effects (p >0.05).

Additional self-report measures, Emotional Breakthrough Inventory (EBI; 9), Psychological Insight Scale (PIS; 10), and Challenging Experience Questionnaire (CEQ, 11), did not significantly moderate antidepressant response (p>0.05).

From the Intensity Rating (IR) - VAS, both Intensity (“How intense was the psychedelic experience?”) (B = –5.69, p = 0.004) and Meaningfulness (“How meaningful was the psychedelic experience?”) (B = –4.64, p = 0.03) significantly predicted greater symptom improvement. A one standard deviation increases in VAS-rated intensity predicted a 5.69-point reduction in MADRS, while higher perceived meaningfulness was associated with a 4.64-point reduction. Other VAS items, including those related to fear or ego dissolution, were not significantly associated with outcome.

Overall, these findings suggest that select dimensions of the acute DMT experience, particularly unity, ego dissolution, and perceived intensity or meaning, may play a role in

shaping clinical outcomes. However, these analyses were exploratory, and further studies with larger samples are needed to replicate and clarify these effects.

Below we additionally report exploratory correlational analyses between different acute measures and changes in depressive symptoms at 2 weeks, containing R and uncorrecred p values.

Figure S6: Exploratory correlational analyses between subjective measures of the acute psychedelic experience and changes in depressive symptoms at 2 weeks containing R and uncorrected p values.

3.11. Supplementary analyses on possible effects of antidepressant washout, prior experience with psychedelics and preparation (set, setting, therapeutic rapport and intention) on antidepressant effects

3.11.1. Effects of antidepressant washout on treatment outcomes

We had 8 participants in the sample who were tapered off antidepressants (see Table 1). To examine whether washout status influenced treatment outcomes, we ran an ANCOVA with Day-14 MADRS as the dependent variable, baseline MADRS as a covariate, and treatment (placebo vs DMT), washout status, and their interaction as factors. The model showed a significant overall treatment effect, but neither the main effect of washout nor the interaction with treatment reached significance. The sample size of the washout group is small, so results should be interpreted cautiously, but these findings suggest that antidepressant washout did not affect clinical response.

	**Sum Sq**	**Df**	**F value**	**Pr(>F)**
baseline	836.6	1	9.61	0.0043 **
Tx	442.9	1	5.09	0.0318 *
AD washout	5.5	1	0.06	0.8038
**Tx × washout**	**1.8**	**1**	**0.02**	**0.8874**
Residual	2523.4	29		

Table S10: Effects of antidepressant washout ANCOVA

3.11.2. Effects of prior psychedelic use on treatment outcomes

Eleven participants reported prior use (5 in the placebo arm and 6 in the DMT arm). To test whether prior use influenced outcomes, we ran an ANCOVA with Day-14 MADRS as the dependent variable, baseline MADRS as a covariate, and treatment (placebo vs DMT), prior use, and their interaction as factors. The model showed a significant overall treatment effect, while neither the main effect of prior use nor the interaction with treatment were significant. This suggests no evidence that prior psychedelic use moderated clinical response, though the subgroup size is limited and results should be considered exploratory.

	**Sum Sq**	**Df**	**F value**	**Pr(>F)**
baseline	931.3	1	11.94	0.0017 **
Tx	488.2	1	6.26	0.0182 *
PriorUse	245.9	1	3.15	0.0862 .

	Sum Sq	Df	F value	Pr(>F)
Tx × PriorUse	23.5	1	0.30	0.5873
Residual	2261.2	29		

Table S11: Effects of prior psychedelic use ANCOVA

3.11.3. Effects of set, setting, rapport, and intention on treatment outcomes

The full sample was used to test the moderating influence of Psychedelic Predictor Scale subscales (PPS; 12) on antidepressant response two weeks following participants' first active DMT dose. As justified by the absence of differences in MADRS change between individuals who received their first active dose after a placebo (PA group) versus those without an initial placebo (AA group), data were consolidated such that Day 1 and Day 14 MADRS scores were used for AA participants, Day 14 and Day 28 scores for PA participants, and Day 1 and Day 15 PPS subscale scores for AA and PA participants, respectively.

Correlations were then calculated between MADRS change scores and each PPS subscale. No significant associations were observed for set ($P = 0.799$, $R^2 < 0.01$), setting ($P = 0.649$, $R^2 < 0.01$), rapport ($P = 0.633$, $R^2 < 0.01$), or intentions ($P = 0.116$, $R^2 = 0.08$). These results indicate that, within the limits of statistical power, none of the PPS dimensions were reliably associated with clinical outcome. Below are displayed these exploratory correlations.

Figure S7: Exploratory correlational analyses between different elements of psychedelic preparation and changes in depressive symptoms at 2 weeks containing R and uncorrected p values.

3.11.4. Adjustment for baseline depression duration

In response to reviewer feedback, we also conducted an additional exploratory ANCOVA to examine whether the duration of depressive illness influenced symptom trajectories. We added Years of depression as a covariate to the mixed model to control for potential effects of illness chronicity on changes in MADRS scores.

The covariate was not significant ($p = 0.46$), and all primary effects, including the Group × Time interaction, remained unchanged. This indicates that baseline illness duration did not meaningfully confound treatment-related differences or overall symptom improvement across visits.

These findings support the robustness of the original model, showing that the observed group effects persist even after adjusting for individual variability in chronicity of depression.

Effect	Sum Sq	Mean Sq	NumDF	DenDF	F value	Pr(>F)
Group	4.40	4.43	1.00	31.98	0.08	0.78
Time	4099.70	819.95	5.00	150.44	14.98	6.37e-12
Years_dep	30.40	30.35	1.00	32.77	0.55	0.46
Group:Time	801.10	160.22	5.00	150.47	2.93	0.01

Table S12: Effects of baseline depression ANCOVA

3.12. Imputation analyses for handling missing data

Because the mixed model for repeated measures (MMRM) assumes data are missing at random, a worst-case scenario sensitivity analysis was conducted to evaluate robustness against potential non-random missingness, such as attrition due to lack of efficacy or adverse events. For each participant and visit, missing MADRS values were conservatively imputed as follows

- In the placebo-active (PA) group, each missing value was replaced with the participant's best observed score minus 2 points (artificially improving outcomes).
- In the active-active (AA) group, each missing value was replaced with the participant's worst observed score plus 2 points (artificially worsening outcomes).

This approach simulates an unfavourable attrition pattern for the AA group. No data were missing at the primary endpoint (Day 14), so the analysis focused on the open label phase. Results from the worst-case model (Table below) showed that the main effect **of** Time remained highly significant, confirming a sustained reduction in MADRS scores across visits. The LMM analysis on follow-up data in the open label phase showed a comparable reduction in depressive symptoms, with no significant differences between the two groups, mirroring the main results.

Term	Estimate	Std_Error	df	t_value	p_value
(Intercept)	25.52	2.48	85.39	10.27	<0.001
PA group estimates					
Day 22	-10.70	2.48	160	-4.30	<0.001

Day 29	-10.58	2.48	160	-4.26	<0.001
Day 45	-12.94	2.48	160	-5.207	<0.001
Day 105	-14.52	2.48	160	-5.846	<0.001
Between condition differences between AA group and PA group					
GroupAA:Day 22	-0.35	3.5147	160	-0.1	0.9201
GroupAA:Day 29	-2.47	3.5147	160	-0.703	0.4831
GroupAA:Day 45	0.76	3.5147	160	0.218	0.8280
GroupAA:Day 105	6.47	3.5147	160	1.841	0.0675

Table S13: Imputation analysis

3.13. Safety Results

There were no clinically significant physical examination findings, laboratory variables, vital signs, or ECGs.

3.13.1. Adverse Events and Serious Adverse Events

A single serious adverse event (SAE), a non-fatal forearm fracture due to a road traffic accident, was recorded in one participant, while another participant self-withdrew from the study following a viral infection. Neither event was considered related to DMT treatment. Apart from the single SAE, all other treatment-emergent adverse events (TEAEs) were mild to moderate in severity, and while some required drug treatment or follow-up with a psychiatrist, they did not interfere with the overall conduct of the trial.

The following table (Table S14:) shows all TEAEs (both deemed related and unrelated to treatment).

System organ class<br>Preferred term	PA Group		AA Group		All participants (N=34)
	Stage 1	Stage 2	Stage 1	Stage 2	
	Placebo (N=17)	DMT after placebo (N=16)	DMT (N=17)	DMT after active (N=13)	
	n (%) [number of TEAEs]				
**Total**	**11 (64.7)**	**14 (87.5)**	**16 (94.1)**	**6 (46.2)**	**33 (97.1)**
**General disorders and administration site conditions**	**4 (23.5)**	**6 (37.5)**	**10 (58.8)**	**1 (7.7)**	**20 (58.8)**
Infusion site pain	3 (17.6) [3]	6 (37.5) [6]	6 (35.3) [6]	1 (7.7) [1]	15 (44.1) [16]
Chest pain	–	1 (6.3) [1]	1 (5.9) [1]	–	2 (5.9) [2]
Catheter site related reaction	–	–	1 (5.9) [1]	–	1 (2.9) [1]
Drug withdrawal syndrome (caffeine)*	–	–	1 (5.9) [1]	–	1 (2.9) [1]
Influenza like illness	–	–	1 (5.9) [1]	–	1 (2.9) [1]
Injection site pain	1 (5.9) [1]	–	–	–	1 (2.9) [1]
**Psychiatric disorders**	**3 (17.6)**	**6 (37.5)**	**3 (17.6)**	**2 (15.4)**	**12 (35.3)**
Anxiety	–	4 (25.0) [4]	2 (11.8) [2]	1 (7.7) [1]	7 (20.6) [7]
Restlessness	1 (5.9) [1]	1 (6.3) [1]	1 (5.9) [1]	–	3 (8.8) [3]
Insomnia	1 (5.9) [1]	1 (6.3) [2]	–	–	2 (5.9) [3]
Sleep disorder	–	2 (12.5) [2]	–	–	2 (5.9) [2]
Depressed mood	1 (5.9) [1]	–	–	–	1 (2.9) [1]
Depression	–	1 (6.3) [1]	–	–	1 (2.9) [1]
Emotional distress	–	1 (6.3) [1]	–	–	1 (2.9) [1]
Middle insomnia	–	–	–	1 (7.7) [1]	1 (2.9) [1]
Pseudohallucination	–	–	1 (5.9) [1]	–	1 (2.9) [1]
Suicidal ideation	1 (5.9) [1]	–	–	–	1 (2.9) [1]
**Nervous system disorders**	**2 (11.8)**	**8 (50.0)**	**5 (29.4)**	**2 (15.4)**	**14 (41.2)**
Headache	2 (11.8) [2]	7 (43.8) [8]	3 (17.6) [3]	1 (7.7) [1]	12 (35.3) [14]
Disturbance in attention	–	–	1 (5.9) [1]	–	1 (2.9) [1]
Dizziness	–	1 (6.3) [1]	–	–	1 (2.9) [1]
Paraesthesia	–	–	1 (5.9) [1]	–	1 (2.9) [1]
Sciatica	–	–	–	1 (7.7) [1]	1 (2.9) [1]
Somnolence	1 (5.9) [1]	–	–	–	1 (2.9) [1]

System organ class<br>Preferred term	PA Group		AA Group		All participants (N=34)
	Stage 1	Stage 2	Stage 1	Stage 2	
	Placebo (N=17)	DMT after placebo (N=16)	DMT (N=17)	DMT after active (N=13)	
	n (%) [number of TEAEs]				
**Gastrointestinal disorders**	**1 (5.9)**	**3 (18.8)**	**3 (17.6)**	**–**	**7 (20.6)**
Nausea	1 (5.9) [1]	3 (18.8) [3]	3 (17.6) [3]	–	7 (20.6) [7]
Abdominal discomfort	–	1 (6.3) [1]	–	–	1 (2.9) [1]
**Infections and infestations**	**1 (5.9)**	**3 (18.8)**	**2 (11.8)**	**1 (7.7)**	**7 (20.6)**
COVID-19	–	2 (12.5) [2]	–	–	2 (5.9) [2]
Viral infection	1 (5.9) [1]	1 (6.3) [1]	–	–	2 (5.9) [2]
Nasopharyngitis	–	–	1 (5.9) [1]	–	1 (2.9) [1]
Rhinitis	–	–	1 (5.9) [1]	–	1 (2.9) [1]
Viral tonsilitis	–	–	–	1 (7.7) [1]	1 (2.9) [1]
**Musculoskeletal and connective tissue disorders**	–	**2 (12.5)**	**2 (11.8)**	–	**4 (11.8)**
Muscle tightness	–	1 (6.3) [1]	1 (5.9) [2]	–	2 (5.9) [3]
Pain in extremity	–	1 (6.3) [1]	1 (5.9) [1]	–	2 (5.9) [2]
**Respiratory, thoracic, and mediastinal disorders**	**2 (11.8)**	**1 (6.3)**	**–**	**1 (7.7)**	**4 (11.8)**
Oropharyngeal pain	1 (5.9) [1]	–	–	1 (7.7) [1]	2 (5.9) [2]
Cough	–	1 (6.3) [1]	–	–	1 (2.9) [1]
Nasal congestion	1 (5.9) [1]	–	–	–	1 (2.9) [1]
**Injury, poisoning, and procedural complications**	**1 (5.9)**	**–**	**–**	**1 (7.7)**	**2 (5.9)**
Forearm fracture	–	–	–	1 (7.7) [1]	1 (2.9) [1]
Scratch	1 (5.9) [1]	–	–	–	1 (2.9) [1]
**Vascular disorders**	**–**	**–**	**2 (11.8)**	**–**	**2 (5.9)**
Hypertension	–	–	1 (5.9) [1]	–	1 (2.9) [1]
Pallor	–	–	1 (5.9) [1]	–	1 (2.9) [1]
**Ear and labyrinth disorders**	**–**	**–**	**–**	**1 (7.7)**	**1 (2.9)**
Tinnitus	–	–	–	1 (7.7) [1]	1 (2.9) [1]
**Eye disorders**	**–**	**–**	**1 (5.9)**	**–**	**1 (2.9)**
Visual snow syndrome	–	–	1 (5.9) [1]	–	1 (2.9) [1]

**System organ class**<br>Preferred term	**PA Group**		**AA Group**		**All participants (N=34)**
	**Stage 1**	**Stage 2**	**Stage 1**	**Stage 2**	
	**Placebo (N=17)**	**DMT after placebo (N=16)**	**DMT (N=17)**	**DMT after active (N=13)**	
	**n (%) [number of TEAEs]**				
**Metabolism and nutrition disorders**	**1 (5.9)**	**–**	**–**	**–**	**1 (2.9)**
Lactose intolerance	1 (5.9) [1]	–	–	–	1 (2.9) [1]
**Skin and subcutaneous tissue disorders**	**–**	**1 (6.3)**	**–**	**–**	**1 (2.9)**
Night sweats	–	1 (6.3) [1]	–	–	1 (2.9) [1]

N: total number of participants; n: number of participants with a TEAE; TEAE: treatment-emergent adverse event. Participants with ≥ 1 TEAE are counted only once per system organ class and preferred term.

* Drug withdrawal syndrome was recorded in 1 participant, and was described as vomiting induced by caffeine withdrawal.

Table S14: All Treatment-emergent adverse events

3.13.2. Vital Signs

Vital signs were collected at the following timepoints:

1. Blood pressure:
   - Day 1 (Visit 2) and Day 15 (Visit 3): predose and at 12, 60 and 240 min after dosing.
   - Day 2 (Visit 2) and Day 16 (Visit 3): before discharge.
2. Heart rate:
   - Day 1 (Visit 2) and Day 15 (Visit 3): predose and at 12, 60 and 240 min after dosing.
   - Day 2 (Visit 2) and Day 16 (Visit 3): before discharge.
3. Tympanic temperature:
   - Day 1 (Visit 2) and Day 15 (Visit 3): predose and at 60 min after dosing.
   - Day 2 (Visit 2) and Day 16 (Visit 3): before discharge.

There were DMT-related changes from baseline in mean systolic and diastolic blood pressure, and pulse rate, at the 12 min postdose timepoint; as seen in healthy participants in Part A (James et al, 2024), those were not sustained to 1 and 4 h postdose.

At the 12 min postdose timepoint, mean changes from baseline in blood pressure (systolic 20.9–24.5 mm Hg; diastolic 14.5–15.8 mm Hg) were similar after DMT dosing, regardless of treatment sequence.

Note that changes from baseline after dosing in Stage 2 were calculated using Stage 1 predose values as baseline. Those change from baseline values may therefore not be completely representative of the participants' baseline on the Stage 2 dosing day.

Postdose vital signs of PCI were recorded in:

- 5 (29.4%) participants who received placebo in Stage 1, and 10 (62.5%) who then received DMT in Stage 2; and
- 10 (58.8%) participants who received DMT in Stage 1, and 9 (69.2%) who then received a second dose in Stage 2.

There was further evidence of a possible relationship between DMT and raised blood pressure and pulse rate, as evidenced below. However, the findings do not demonstrate increased incidence of values of PCI after more than 1 dose of DMT.

Postdose systolic blood pressures of PCI were recorded in:

- 3 (17.6%) participants who received placebo in Stage 1, and 9 (56.3%) who then received DMT in Stage 2; and
- 10 (58.8%) participants who received DMT in Stage 1, and 8 (61.5%) who then received a second dose in Stage 2.
- Postdose diastolic blood pressures of PCI were recorded in:
- 1 (5.9%) participant who received placebo in Stage 1, and 6 (37.5%) who then received DMT in Stage 2; and
- 10 (58.8%) participants who received DMT in Stage 1, and 8 (61.5%) who then received second dose in Stage 2.

Postdose pulse rates of PCI were recorded in:

- 5 (31.3%) participants who received DMT in Stage 2 (no participant had a pulse rate of PCI after receiving placebo); and
- 4 (23.5%) participants who received DMT in Stage 1, and 2 (15.4%) participants who then received a second dose in Stage 2.

Instances of blood pressure of PCI also coincided with relevant AEs in 2 participants, as follows.

- One AA participant had predose low systolic blood pressure (84 mm Hg) and pulse rate (38 beats/min) that were recorded as a predose AE of vasovagal attack.
- One AA participant had raised blood pressure (systolic 169 mm Hg; diastolic 123 mm Hg) and pulse rate (116 beats/min) that were recorded as a TEAE of hypertension that started at 13 min after their first dose and lasted 29 min. The participant also had similarly raised blood pressure (systolic 155 mm Hg; diastolic 96 mm Hg) at the same timepoint after their second dose (no associated AE was recorded).

There were no conclusive differences among treatment groups with respect to mean body temperature, nor incidence of temperatures of PCI, recorded in:

- 2 (11.8%) participants who received placebo in Stage 1, and 1 (6.3%) who then received DMT in Stage 2; and
- 2 (11.8%) participants who received DMT in Stage 1, and 2 (15.4%) who then received a second dose in Stage 2.

									Change from Baseline					
Variable (units)	Treatment	Planned Relative Time	n	Mean	SD	Median	Min	Max	n	Mean	SD	Median	Min	Max
Systolic Blood Pressure (mmHg)	Placebo (N=17)	Day 1, Pre-dose	17	117.6	12.02	120.0	95	134						
		Day 1, 12 min	17	124.5	12.21	124.0	105	144	17	6.9	10.33	8.0	-15	29
		Day 1, 60 min	17	120.8	13.84	118.0	102	149	17	3.2	8.63	4.0	-17	16
		Day 1, 240 min	16	121.2	15.54	121.5	97	145	16	3.7	9.09	1.5	-5	30
		Day 2	17	116.6	11.80	118.0	93	137	17	-1.1	8.37	-2.0	-13	16
	21.5 mg SPL026 (Stage 2 after Placebo) (N=16)	Day 15, Pre-dose	16	114.9	8.99	114.5	102	131	16	-1.9	10.56	1.0	-19	11
		Day 15, 12 min	16	141.3	17.64	142.5	105	179	16	24.5	16.35	22.5	-12	57
		Day 15, 60 min	16	120.3	11.42	122.0	99	142	16	3.5	11.45	3.0	-11	36
		Day 15, 240 min	16	118.8	11.39	115.0	102	135	16	2.0	10.76	3.5	-25	24
		Day 16	16	119.9	12.22	118.5	98	140	16	3.2	9.47	1.0	-10	28
	21.5 mg SPL026 (Stage 1) (N=17)	Day 1, Pre-dose	17	117.3	12.82	115.0	94	139						
		Day 1, 12 min	17	138.2	19.93	142.0	106	173	17	20.9	16.13	16.0	-5	50
		Day 1, 60 min	17	123.3	15.96	124.0	96	153	17	6.0	8.48	5.0	-13	22
		Day 1, 240 min	16	119.9	13.74	120.0	95	141	16	2.5	7.54	2.0	-11	19
		Day 2	17	116.6	10.41	117.0	95	134	17	-0.7	10.87	1.0	-20	15
	21.5 mg SPL026 (Stage 2 after Active) (N=13)	Day 15, Pre-dose	13	114.4	14.76	111.0	88	137	13	-5.2	9.14	-3.0	-24	7
		Day 15, 12 min	12	140.9	14.79	141.5	109	164	12	22.3	16.24	23.0	-7	46
		Day 15, 60 min	13	119.6	12.89	118.0	100	145	13	0.0	7.97	1.0	-13	10
		Day 15, 240 min	13	119.6	15.40	123.0	92	147	13	0.0	8.77	-4.0	-12	16
		Day 16	13	118.4	12.35	118.0	90	135	13	-1.2	5.57	1.0	-12	5

Table S15: Summary of Vital Signs – Systolic Blood Pressure

									Change from Baseline					
Variable (units)	Treatment	Planned Relative Time	n	Mean	SD	Median	Min	Max	n	Mean	SD	Median	Min	Max
Diastolic Blood Pressure (mmHg)	Placebo (N=17)	Day 1, Pre-dose	17	71.0	7.47	70.0	61	86						
		Day 1, 12 min	17	73.5	9.19	73.0	59	92	17	2.5	6.40	4.0	-8	12
		Day 1, 60 min	17	72.2	9.42	71.0	56	88	17	1.2	8.00	0.0	-14	14
		Day 1, 240 min	16	71.5	8.62	69.5	58	89	16	0.8	4.43	0.0	-6	10
		Day 2	17	72.2	7.29	72.0	60	83	17	1.2	6.54	0.0	-10	15
	21.5 mg SPL026 (Stage 2 after Placebo) (N=16)	Day 15, Pre-dose	16	68.5	5.35	68.0	57	78	16	-1.6	6.48	0.0	-16	14
		Day 15, 12 min	16	85.6	11.89	85.0	64	101	16	15.5	12.36	15.5	-19	31
		Day 15, 60 min	16	72.4	9.33	71.5	54	87	16	2.3	6.88	2.0	-10	13
		Day 15, 240 min	16	70.1	9.16	68.0	59	83	16	0.1	10.14	0.5	-21	19
		Day 16	16	71.5	10.07	70.0	55	89	16	1.4	8.94	1.0	-14	18
	21.5 mg SPL026 (Stage 1) (N=17)	Day 1, Pre-dose	17	71.0	8.65	67.0	58	87						
		Day 1, 12 min	17	85.5	15.22	86.0	61	123	17	14.5	11.74	10.0	1	42
		Day 1, 60 min	17	76.2	13.14	72.0	55	97	17	5.2	6.93	6.0	-6	20
		Day 1, 240 min	16	69.8	10.57	68.5	54	86	16	-1.4	4.87	-1.5	-13	7
		Day 2	17	71.2	9.52	69.0	54	93	17	0.2	6.30	0.0	-11	10
	21.5 mg SPL026 (Stage 2 after Active) (N=13)	Day 15, Pre-dose	13	68.8	9.96	65.0	54	91	13	-3.8	7.44	-6.0	-14	13
		Day 15, 12 min	12	87.6	15.54	88.5	57	118	12	15.8	13.31	12.0	-1	51
		Day 15, 60 min	13	75.5	12.86	74.0	58	97	13	2.9	5.28	3.0	-5	14
		Day 15, 240 min	13	68.8	8.42	68.0	58	89	13	-3.8	4.76	-4.0	-12	6
		Day 16	13	72.3	10.83	70.0	55	97	13	-0.3	10.05	-2.0	-14	25

Table S16: Summary of Vital Signs – Diastolic Blood Pressure

									Change from Baseline					
Variable (units)	Treatment	Planned Relative Time	n	Mean	SD	Median	Min	Max	n	Mean	SD	Median	Min	Max
Heart Rate (beats/min)	Placebo (N=17)	Day 1, Pre-dose	17	64.7	10.31	60.0	51	86						
		Day 1, 12 min	12	69.7	9.16	69.5	55	83	12	4.0	10.17	3.0	-9	26
		Day 1, 60 min	17	69.9	11.81	71.0	48	94	17	5.2	10.83	3.0	-13	24
		Day 1, 240 min	16	65.6	8.14	68.5	52	76	16	0.6	8.52	-0.5	-11	17
		Day 2	17	63.9	9.33	63.0	45	79	17	-0.8	9.43	-1.0	-15	14
	21.5 mg SPL026 (Stage 2 after Placebo) (N=16)	Day 15, Pre-dose	16	63.9	10.41	62.0	52	85	16	0.6	8.07	-0.5	-14	21
		Day 15, 12 min	11	97.7	21.49	99.0	57	127	11	33.9	18.75	46.0	-2	52
		Day 15, 60 min	16	72.3	12.04	72.5	51	93	16	8.9	7.49	7.5	-2	25
		Day 15, 240 min	16	67.6	10.22	69.5	48	80	16	4.2	10.11	4.0	-16	19
		Day 16	16	62.7	9.17	62.0	49	79	16	-0.7	6.96	-0.5	-9	18
	21.5 mg SPL026 (Stage 1) (N=17)	Day 1, Pre-dose	17	65.2	11.88	63.0	43	94						
		Day 1, 12 min	13	88.7	21.00	89.0	54	120	13	22.9	14.86	20.0	-1	53
		Day 1, 60 min	17	71.2	10.77	67.0	58	93	17	6.1	9.46	5.0	-12	22
		Day 1, 240 min	16	66.8	11.53	66.0	41	84	16	1.3	9.60	2.0	-26	13
		Day 2	17	66.2	11.87	69.0	41	89	17	1.0	9.30	1.0	-18	20
	21.5 mg SPL026 (Stage 2 after Active) (N=13)	Day 15, Pre-dose	13	63.8	10.58	62.0	45	78	13	-1.7	8.10	-3.0	-18	15
		Day 15, 12 min	12	89.8	26.57	86.0	50	147	12	23.6	19.20	25.0	-5	69
		Day 15, 60 min	13	67.5	9.85	68.0	56	85	13	2.1	9.60	1.0	-14	17
		Day 15, 240 min	13	66.7	9.58	68.0	50	83	13	1.2	8.40	2.0	-11	15
		Day 16	13	63.0	12.30	65.0	43	86	13	-2.5	9.04	-4.0	-23	12

Table S17: Summary of Vital Signs – Heart Rate

3.13.3. 12-Lead Electrocardiogram

There were no notable changes or differences between treatments with respect to mean 12-lead ECG variables (heart rate, PR, QRS, QT, QTcF) in the study.

There were no QTcF values of PCI in the study.

All abnormal ECG findings were considered by the investigator to be of no clinical significance.

3.13.4. Injection Site Reactions

The following participants had mild pain and/or tenderness when measured at the 1 h postdose timepoint after their first (Day 1) and/or second doses (Day 15).

Mild pain was measured in:

- 2 (11.8%) participants who received placebo in Stage 1, and 1 (6.3%) who then received DMT in Stage 2; and
- 1 (5.9%) participant who received DMT in Stage 1, but none after their second dose in Stage 2.

Mild tenderness was measured in 3 (17.6%) participants who received placebo in Stage 1 only.

No participant had erythema or induration at the 1 h postdose timepoint at either timepoint.

There were no haematology values of potential clinical importance (PCI). The following participants had isolated double-flagged clinical chemistry or coagulation values at 4 h postdose (unless otherwise stated).

3.13.5. Subjective Tolerability

Tolerability was determined by asking the participant during the postdose integration session: “Do you wish you had not gone through that experience?”. All participants answered ‘No’ to this question except one participant answered ‘Yes’ after their second dose of DMT (worried that ‘it had undone [their] first experience’).

3.13.6. Clinical Chemistry and Coagulation

3 participants with MDD had double-flagged glucose (reference interval 2.8–8.0 mmol/L) after DMT dosing:

- One PA participant had 8.1 mmol/L glucose after their only DMT dose (in Stage 2).
- One A- participant had 8.2 mmol/L glucose after their only DMT dose (in Stage 1).
- One AA participant had 9.5 mmol/L glucose after their second DMT dose (in Stage 2).

One PA participant had 4.2 mmol/L triglycerides (reference interval 0.5–3.7 mmol/L) after their only DMT dose (in Stage 2).

One AA participant had 670 IU/L creatine kinase (reference interval ≤ 641 IU/L) about 14 days after their second DMT dose (repeat sample; in Stage 2).

7 participants with MDD had double-flagged aPTT values (reference interval 28.0–42.0 sec) at 240 min after DMT dosing:

- One AA participant had 25.6 sec aPTT after their second DMT dose (in Stages 2).
- One AA participant had 23.6 sec aPTT after their first DMT dose (in Stage 1).
- One PA participant had 48.5 sec aPTT after their only DMT dose (about 20 days after dosing [Day 15, 240 min postdose repeat sample]). This value was not concomitant with any other relevant abnormalities or concerning physical features, in the opinion of the investigator.
- One PA participant had 25.7 sec aPTT after their only DMT dose.
- One PA participant had 26.4 sec aPTT after their only DMT dose.
- Two participants had double-flagged aPTT values of 24.7 and 23.3 sec, respectively, at 240 min after placebo dosing.

No laboratory value in the study was considered by the investigator to be clinically significant.

3.13.7. Suicidal Ideation

17 participants with MDD had a total score ≥ 1 on at least 1 occasion on both the shortened and full BSS.

During the study, there were no important or concerning changes in BSS score recorded by the investigator. However, the following findings on the full BSS were of note.

- One PA participant scored 0 at screening, 8 on Days –1 and 8, and 7 on Day 14 (after Stage 1 dosing). They also reported a TEAE of (worsening) suicidal ideation on Day 14 (duration: 92 days; considered unlikely to be related to the study treatment). During Stage 2, they reported a TEAE of emotional distress (severe disappointment reaction) on Day 15 (onset: 55 min after receiving DMT; duration: 91 day; possibly related to the study treatment). In their Stage 2 postdose integration session, they expressed that they were very disappointed not to have had a deeper experience. Subsequently, their full BSS total score was 6 on Days 22, 29, and 45. However, they were followed-up with psychiatric review and their final BSS score was 0 by follow-up (Day 105).
- One PA participant scored 20 and 14 at screening and Day –1, respectively. This fell to 12 on Days 8 and 14 (after Stage 1 dosing), increased to 14 on Day 22 (after Stage 2 dosing), but fell again to 11 by follow up (Day 105). They had no concomitant TEAEs of suicidality, and their dosing experiences were adequately tolerated (as determined from their postdose integration sessions).
- One PA participant scored 11 and 10 at screening and Day –1, respectively, but this fell to 2 on Days 8 and 14 (after Stage 1 dosing). Their score increased to 4 on Day 22 (after Stage 2 dosing), but fell to 1 on Day 29 and 0 on Days 45 and at follow-up (Day 105). They had no concomitant TEAEs of suicidality, and their dosing experiences were well tolerated (as determined from their postdose integration sessions).

6 participants had a change from baseline scores (relative to Day –1) of ≥ ±2 at 1 or more timepoints. Of those participants, only one participant had a higher score at the end of the study than at predose (an increase from 1 to 4). All other participants had change from baseline scores (relative to Day –1) of either 0 or ±1.

All other participants had a total score of no higher than 5 at any timepoint on the full BSS.

Treatment Group	Time Point (Days)	n	Mean	SD	Min	Max
Placebo (Stage 1) (N=17)	Day -1	17	2.5	4.26	0	14
	Day 8	17	1.6	3.37	0	12
	Day 14	16	1.4	3.34	0	12
21.5 mg DMT after placebo (Stage 2) (N=16)	Day 22	15	1.6	3.87	0	14
	Day 29	15	1.3	3.10	0	11
	Day 45	15	1.1	2.90	0	10
	Day 105	13	1.0	3.06	0	11
21.5 mg DMT (Stage 1) (N=17)	Day -1	17	0.5	0.80	0	2
	Day 8	16	0.2	0.54	0	2
	Day 14	17	0.2	0.56	0	2
21.5 mg DMT after active (Stage 2) (N=13)	Day 22	13	0.0	0.00	0	0
	Day 29	13	0.0	0.00	0	0
	Day 45	12	0.2	0.39	0	1
	Day 105	13	0.2	0.38	0	1

Table S18: Summary of Beck Scale for Suicidal Ideation (BSS) Full

4. Supplementary References

1. The Medicines for Human Use (Clinical Trials) Regulations 2004 (SI 2004 No. 1031), as amended by The Medicines for Human Use (Clinical Trials) Amendment Regulations 2006 (SI 2006 No. 1928).
2. The Medicines for Human Use (Clinical Trials) Amendment (No. 2) Regulations 2006 (SI 2006 No. 2984), The Medicines for Human Use (Clinical Trials) and Blood Safety and Quality (Amendment) Regulations 2008 (SI 2008 No. 941).
3. The Medicines for Human Use (Miscellaneous Amendments) Regulations 2009 (SI 2009 No. 1164), and The Medicines for Human Use (Clinical Trials) (Amendment) (EU Exit) Regulations 2019 (SI 2019 No. 744).
4. The Human Medicines Regulations 2012 (SI 2012 No. 1916), with subsequent amendments, including: the Human Medicines (Amendment etc.) (EU Exit) Regulations 2019 (SI 2019 No. 775),the Human Medicines and Medical Devices (Amendment etc.) (EU Exit) Regulations 2019 (SI 2019 No. 1385), and the Human Medicines (Amendment etc.) (EU Exit) Regulations 2020 (SI 2020 No. 1488).
5. Rules Governing Medicinal Products in the European Union, Volume 4 - Good Manufacturing Practice, incorporating Directive 2003/94/EC.
6. Watts, R., & Luoma, J. B. (2020). The use of the psychological flexibility model to support psychedelic-assisted therapy. Journal of Contextual Behavioral Science, 15, 92-102. https://doi.org/10.1016/j.jcbs.2019.12.004
7. Studerus, E., A. Gamma, and F.X. Vollenweider, *Psychometric evaluation of the altered states of consciousness rating scale (OAV).* PloS one, 2010. **5**(8): p. e12412
8. Nour, M.M., et al., *Ego-Dissolution and Psychedelics: Validation of the Ego-Dissolution Inventory (EDI).* Front Hum Neurosci, 2016. **10**: p. 269.
9. . Roseman, L., et al., *Emotional breakthrough and psychedelics: Validation of the Emotional Breakthrough Inventory.* J Psychopharmacol, 2019. **33**(9): p. 1076-1087.
10. 29. Barrett, F.S., et al., *The Challenging Experience Questionnaire: Characterization of challenging experiences with psilocybin mushrooms.* J Psychopharmacol, 2016. **30**(12): p. 1279-1295.
11. Peill, J.M., et al., *Validation of the Psychological Insight Scale: A new scale to assess psychological insight following a psychedelic experience.* J Psychopharmacol, 2022. **36**(1): p. 31-45.
12. Angyus, M., Osborn, S., Haijen, E., Erritzoe, D., Peill, J., Lyons, T., Kettner, H., & Carhart-Harris, R. (2024). Validation of the imperial psychedelic predictor scale. *Psychological medicine*, *54*(12), 1–9. Advance online publication.
