# Supplementary material for: A short-acting psychedelic intervention for major depressive disorder: a phase IIa randomized placebo-controlled trial
Source: Nat Med. 2026 Feb 16;32(2):591–8. doi: 10.1038/s41591-025-04154-z (PMC12920121; doi:10.1038/s41591-025-04154-z)
Supplement: Supplementary file 2 — Reporting Summary [file 41591_2025_4154_MOESM2_ESM.pdf]

nature portfolio

Corresponding author(s): Dr David Erritzoe, Mr Tommaso Barba, Dr Ellen James

Last updated by author(s): 4/11/2025

Reporting Summary

Nature Portfolio wishes to improve the reproducibility of the work that we publish. This form provides structure for consistency and transparency in reporting. For further information on Nature Portfolio policies, see our Editorial Policies and the Editorial Policy Checklist.

Statistics

For all statistical analyses, confirm that the following items are present in the figure legend, table legend, main text, or Methods section.

n/a	Confirmed	
☐	☒	The exact sample size (*n*) for each experimental group/condition, given as a discrete number and unit of measurement
☒	☐	A statement on whether measurements were taken from distinct samples or whether the same sample was measured repeatedly
☐	☒	The statistical test(s) used AND whether they are one- or two-sided<br>*Only common tests should be described solely by name; describe more complex techniques in the Methods section.*
☐	☒	A description of all covariates tested
☐	☒	A description of any assumptions or corrections, such as tests of normality and adjustment for multiple comparisons
☐	☒	A full description of the statistical parameters including central tendency (e.g. means) or other basic estimates (e.g. regression coefficient) AND variation (e.g. standard deviation) or associated estimates of uncertainty (e.g. confidence intervals)
☐	☒	For null hypothesis testing, the test statistic (e.g. *F*, *t*, *r*) with confidence intervals, effect sizes, degrees of freedom and *P* value noted<br>*Give P values as exact values whenever suitable.*
☒	☐	For Bayesian analysis, information on the choice of priors and Markov chain Monte Carlo settings
☒	☐	For hierarchical and complex designs, identification of the appropriate level for tests and full reporting of outcomes
☐	☒	Estimates of effect sizes (e.g. Cohen's *d*, Pearson's *r*), indicating how they were calculated

Our web collection on statistics for biologists contains articles on many of the points above.

Software and code

Policy information about availability of computer code

Data collection	Data was collected on an electronic case report form using the Medrio EDC system (latest current version at time of use; 2020-2022).
Data analysis	The prespecified statistical analysis was done using SAS 9.4 The post-hoc analyses were done using version of R current at the time.

For manuscripts utilizing custom algorithms or software that are central to the research but not yet described in published literature, software must be made available to editors and reviewers. We strongly encourage code deposition in a community repository (e.g. GitHub). See the Nature Portfolio guidelines for submitting code & software for further information.

Data

Policy information about availability of data

All manuscripts must include a data availability statement. This statement should provide the following information, where applicable:

- Accession codes, unique identifiers, or web links for publicly available datasets
- A description of any restrictions on data availability
- For clinical datasets or third party data, please ensure that the statement adheres to our policy

The clinical trial data underlying this article are not publicly available due to privacy concerns and restrictions from the trial sponsor (Cybin IRL Ltd). In line with ICMJE guidelines, de-identified participant data will be made available upon reasonable request to the sponsor for the purposes of verifying the results within 1 month of execution of a data sharing agreement. Access will be granted to qualified researchers following review and subject to a data sharing agreement to ensure participant confidentiality and compliance with regulatory requirements.

Research involving human participants, their data, or biological material

Policy information about studies with human participants or human data. See also policy information about sex, gender (identity/presentation), and sexual orientation and race, ethnicity and racism.

Reporting on sex and gender	Findings apply to males and females. The tables use the term sex rather than gender, but this was determined based on self-reporting and may reflect gender identity rather than sex assigned at birth. Overall numbers of males and females has been reported but sex/gender-based analyses have not been performed due to overall low sample size.
Reporting on race, ethnicity, or other socially relevant groupings	The race/ethnicity categories used followed the FDA's recommendations which are: American Indian or Alaska Native; Asian; Black or African American; Native Hawaiian or Other Pacific Islander; and White. Participants were allowed to self-report and could also designate themselves as multiracial. Ethnicity was separated from race, with the categories: Hispanic/Latino or Not Hispanic/Latino. These categories were not used for any inferences or analyses. Summary information is presented in Table 1 in the main paper.
Population characteristics	Participants had a mean age of 32.8 years (range 21–53) and were mostly male (71%) and White (88%). Average BMI was 24.8 kg/m². Mean duration of depression was 10.4 years, with baseline MADRS and HAM-D scores indicating moderate-to-severe depression. Prior psychedelic use was reported by 11 participants (32%), and eight had recently discontinued antidepressants before dosing. See Table 1 in the main paper for full demographic details.
Recruitment	Adults aged 18 or over with a diagnosis of moderate-to-severe MDD and a history of at least two prior unsuccessful treatment attempts (pharmacological or/and psychotherapeutic) were recruited formally through databases held by the clinical trial sites, informally through social media, and through other sources, which directed participants to a recruitment website. Possible biases include self-selection through online and social media recruitment, which may have favored participants more open or motivated toward novel psychedelic treatments.
Ethics oversight	The trial protocol was reviewed and approved by the UK Medicines and Healthcare products Regulatory Agency (MHRA) and the London–Brent Research Ethics Committee (REC). All participants provided written informed consent. The trial is registered on clinicaltrials.gov (NCT04673383) and ISRCTN (ISRCTN63465876), where the clinical study report synopsis is available.

Note that full information on the approval of the study protocol must also be provided in the manuscript.

Field-specific reporting

Please select the one below that is the best fit for your research. If you are not sure, read the appropriate sections before making your selection.

☒ Life sciences ☐ Behavioural & social sciences ☐ Ecological, evolutionary & environmental sciences

For a reference copy of the document with all sections, see nature.com/documents/nr-reporting-summary-flat.pdf

Life sciences study design

All studies must disclose on these points even when the disclosure is negative.

Sample size	The sample size calculation was based on a two-sided, two-sample t-test with equal variance at a significance level of 0.05 and a 1:1 allocation ratio, using data from Palhano-Fontes et al. (2019), an existing study investigating the antidepressant effects of the DMT containing brew Ayahuasca. A sample size of 28 to 36 participants provided 80% to 90% power to detect a 12.5-point mean difference in MADRS score change from baseline.
Data exclusions	No data were excluded from the main analysis, except for one participant's Day 105 MADRS and BSS data which was collected 51 days outside of the deviation window; and 1 participant's Day 8 questionnaires that were lost and re-completed at an unknown time outside of the deviation window.
Replication	No independent replication has been completed yet. This was a phase 2a randomized, blinded Stage-1 study with an open-label Stage-2. We followed a written protocol and SOPs for dosing, timing, and MADRS assessments. Raters were trained and calibrated. All analyses were run by professional statisticians from locked, version-controlled scripts. Sensitivity analyses requested in the review process, including worst-case imputations, gave consistent conclusions.
Randomization	In Stage 1 of the study, participant numbers were allocated to blinded treatments (active or placebo) according to a 1:1 randomisation schedule prepared by an independent HMR statistician, using SAS statistical analysis software.
Blinding	Due to the noticeable psychedelic effects of DMT fumarate, it was likely that both participants and investigators could determine whether a participant had received the active drug or placebo. Despite this, Stage 1 of the study adhered to double-blind principles, while Stage 2 was conducted as an open-label phase. MADRS assessments during both stages were carried out by an independent assessor who remained blinded and was not present during dosing or integration sessions.<br>The trial medication was repackaged and relabelled by the HMR Pharmacy according to the randomisation schedule. Active and placebo treatments were labelled in a way that made them indistinguishable. If the expiry dates of the placebo and active treatments differed, the labels used the earlier expiry date for both treatments to avoid identification. Each participant's treatment was assigned a unique code number traceable to the medication batch number.<br>The active and placebo treatments were similar in appearance, with only slight differences in colour. To further maintain blinding, the HMR

Pharmacy prepared syringes with the correct dosage volumes and obscured the contents with tape, ensuring that investigators could not discern any differences between the solutions. All participants in a group received the same volume of treatment to ensure consistency.
A sealed copy of the randomisation code was stored securely in the HMR Pharmacy, with another copy held by the bioanalytical laboratory. For emergencies, the investigator was provided with sealed envelopes containing treatment allocations for individual participants. These envelopes were stored in the trial master file and were readily accessible to clinical staff if needed.
While emergency procedures for unblinding were in place, no unblinding was required during the trial. Investigators, study psychiatrists, therapists, the Medical Monitor, and the Clinical Monitor remained blinded throughout the study.

Reporting for specific materials, systems and methods

We require information from authors about some types of materials, experimental systems and methods used in many studies. Here, indicate whether each material, system or method listed is relevant to your study. If you are not sure if a list item applies to your research, read the appropriate section before selecting a response.

Materials & experimental systems

n/a	Involved in the study
☒	☐ Antibodies
☒	☐ Eukaryotic cell lines
☒	☐ Palaeontology and archaeology
☒	☐ Animals and other organisms
☐	☒ Clinical data
☒	☐ Dual use research of concern
☒	☐ Plants

Methods

n/a	Involved in the study
☒	☐ ChIP-seq
☒	☐ Flow cytometry
☒	☐ MRI-based neuroimaging

Clinical data

Policy information about clinical studies
All manuscripts should comply with the ICMJE guidelines for publication of clinical research and a completed CONSORT checklist must be included with all submissions.

Clinical trial registration	EudraCT: 2020-000251-13; clinicaltrials.gov: NCT04673383; ISRCTN: ISRCTN63465876
Study protocol	The full protocol has not been made available for reasons of commercial sensitivity.
Data collection	Data was collected at clinical trial sites and at participants homes on paper source documents. The data was entered into an electronic database, then source data verified by the Clinical Monitor and QC'd by the QA team before analysis. Data was collected from first visit (07OCT2021) to last visit (21DEC2022).
Outcomes	Primary and secondary outcome measures were predefined in the clinical trial protocol which was finalised and approved by the MHRA and REC before the first visit took place. The analysis was defined in the statistical analysis plan which was finalised before the clinical trial database was locked. How the measures were assessed is outlined in the methods section of the manuscript.

Plants

Seed stocks	NA
Novel plant genotypes	NA
Authentication	NA
